# Sold a pup? Impact of purchasing practices, owner and dog demographics, and puppy early-life experiences on later canine health outcomes in the UK

**DOI:** 10.1017/awf.2026.10077

**Published:** 2026-04-01

**Authors:** Fiona C. Dale, Dan G. O’Neill, Claire L. Brand, Zoe Belshaw, Bree L. Merritt, Camilla L. Pegram, Rowena M. A. Packer

**Affiliations:** 1 Clinical Science and Services, Royal Veterinary College, United Kingdom; 2 Pathobiology and Population Sciences, Royal Veterinary College, United Kingdom; 3 EviVet Evidence-Based Veterinary Consultancy, United Kingdom

**Keywords:** Animal welfare, canine, dog health, illegal, purchase, veterinary

## Abstract

Despite common assertions that puppies acquired from low-welfare sources (e.g. ‘puppy farms’) and/or sold illegally (e.g. without buyers seeing their puppies’ mother pre-purchase) have poorer future health and welfare, remarkably little evidence supports this. We investigated the impact of puppy early-life risk factors, including owner acquisition behaviours, upon adult dog health outcomes. An online longitudinal survey followed a cohort of n = 985 ‘Pandemic Puppies’ purchased in the UK during 2020 aged < 16 weeks of age as they reached 21 months of age. Owners reported their dogs’ diagnosed health disorders and their expectations vs realities of veterinary costs since a ‘puppyhood’ questionnaire (while ≤ 7 months of age) in 2020. Multivariable modelling investigated risk factors for these outcomes, including early-life health, behaviour, and acquisition-related variables. Most owners (n = 931/985; 94.5%) reported ≥ one health problem in their dog since the 2020 questionnaire. Puppies sold < 6 weeks of age, without their owner having seen the puppy’s mother prior to purchase, or acquired by first-time owners were more likely to have a higher number of health disorders at 21 months old. One-quarter (n = 220/936; 23.5%) of owners had spent more than they expected on veterinary costs since acquiring their puppy, with owners of puppies sold without a microchip more likely to report this. Results suggest that longer-term health outcomes are linked to how and where a puppy is acquired. As many risk factors identified here are already illegal in England, Wales and Scotland, greater enforcement and awareness of this legislation is urgently needed to protect canine welfare.

## Introduction

An estimated 800,000 to 1.3 million puppies are purchased annually in the UK (Maher & Wyatt 2021). Getting a new puppy is typically viewed as a happy time for owners as they look forward to welcoming a new dog into their family, often with high expectations of the benefits they will bring to human members of their households (Costa *et al.* 2023; Belshaw *et al.* 2025a). However, evidence suggests many prospective owners are poorly equipped for successfully navigating the puppy-buying process. While the available time and energy as well as individual circumstances of prospective owners will likely affect their ability to thoroughly research where to optimally source their dog from (Holland 2019), previous studies have found that one-tenth to one-fifth of owners did no research prior to the purchase of their puppy (Packer *et al.* 2017; Kuhl *et al.* 2022; People’s Dispensary for Sick Animals [PDSA] 2024b). Entering the puppy-buying process as a well-informed consumer is of vital importance to animal welfare, due to increasing risks of purchasing puppies from puppy breeders and/or dealers whose prime concern is profit, who supply puppies who are born and raised in low welfare environments (e.g. ‘puppy farms’, illegal importations; Wauthier *et al.* 2018; Packer *et al.* 2021). Recent evidence suggests that only 14.6–19.5% of puppies in the UK come from breeders with a licence, with around 75–80% acquired from other unknown sources, including unlicensed breeders, puppy farms and illegal imports (Naturewatch 2025).

Following the implementation of The Animal Welfare (Licensing of Activities Involving Animals) (England) Regulations 2019 (named Lucy’s Law) in April 2020 (UK Government 2019), the Animal Welfare (Licensing of Activities Involving Animals) (Scotland) Regulations 2021 in September 2021 (UK Government 2021a) and The Animal Welfare (Licensing of Activities Involving Animals) (Wales) Regulations 2021 in September 2021 (UK Government 2021b), it has been illegal to sell a puppy via a third-party, e.g. from a pet shop, or to sell a puppy without the buyer seeing the puppy at the place they were bred and interacting with their mother. The enactment of this legislation was largely driven by an aim to combat welfare issues relating to large-scale dog production by ‘puppy-farms’ both in the UK and abroad which are widely perceived as operating with little evidence of concern for the welfare of the breeding stock or resulting puppies (McMillan 2017). Furthermore, it is illegal in the UK (England, Wales, Scotland and Northern Ireland) to sell any puppy aged under eight weeks (56 days) (UK Government 2013, 2018, 2021a,b). Despite these clear legislative rules that were put in place to protect both puppies and their new owners, many puppy buyers continue to engage in non-recommended purchasing behaviours and risk inadvertently supporting illegal sources. For example, there is evidence that prior to the COVID-19 pandemic, many owners purchased puppies in the UK that were too young for legal sale (e.g. one-quarter of puppies purchased in 2016–2019 were aged under 8 weeks at sale; Kinsman *et al.* 2020), or purchased puppies without their mother present (e.g. 14% in 2019 [Packer *et al.* 2021]; 8.1% in 2016–2019 [Kinsman *et al.* 2020]), or away from their breeder’s property (15% in 2019; Packer *et al.* 2021). Buyers engaging in sales of this nature risk being so-called ‘Petfished’, a term coined by the UK Department for the Environment, Food & Rural Affairs (DEFRA) to describe situations where “*unscrupulous sellers pretend that the puppy they’re selling comes from a happy home, but in reality, the animal may have been bred or kept in poor conditions*” (DEFRA 2021). These low-welfare breeding establishments may be located within or outside of the UK. It is increasingly recognised that the UK demand for puppies is often met by overseas ‘suppliers’ breeding puppies in poor conditions in Central and Eastern Europe which then undergo illegal importation over long routes into the UK (Belshaw & Packer 2025; Belshaw *et al.* 2025b). This process can add further severe welfare impacts to any intrinsic breeding issues (Maher & Wyatt 2021).

There are growing concerns regarding the long-term health and welfare of puppies originating from illegal and/or low-welfare sources. Some evidence already exists regarding negative behavioural outcomes of puppies acquired from such sources, with puppies purchased via pet shops (Jagoe 1994; McMillan *et al.* 2013; Casey *et al.* 2014; Pirrone *et al.* 2016), via puppy farms (Wauthier *et al.* 2018), acquired under 8 weeks of age, or purchased without the mother and father present (Pierantoni *et al.* 2011; Westgarth *et al.* 2012) reported to later exhibit increased levels of problem behaviours as adults. In contrast, the longer-term physical health consequences of acquiring a dog from low-welfare and/or illegal sources, and from owners not following recommended purchasing behaviours have been little studied. To the authors’ knowledge, only one 30 year old study exists that investigated selling characteristics on short-term physical health outcomes. That 1993 study reported that puppies separated from their mother at six weeks of age displayed greater weight loss, disease susceptibility and mortality by six months of age than puppies separated from their mother at 12 weeks old (Slabbert & Rasa 1993). Multiple factors may contribute to any increased risk of physical or behavioural health issues later in life for puppies purchased from low-welfare sources. These factors include that these puppies may be prone to genetic problems (e.g. due to poor breeding choices and lack of health screening; Wauthier *et al.* 2018), receive inadequate veterinary care (e.g. due to deficits in preventative healthcare for both puppies and their mothers; PDSA 2024a), have nutritional deficiencies (e.g. due to poor diet in early developmental stages; Royal Society for the Prevention of Cruelty to Animals [RSPCA] Australia 2024), and are at higher risk of disease transmission (e.g. due to overcrowded and unsanitary living conditions; Dendoncker *et al.* 2018). However, robust evidence to support these concerns is lacking, as breeders producing puppies and dealers trading in puppies reared under these conditions are less likely to allow/engage in formal research of their puppies, and thus proxy measures (e.g. illegal sales indicators, such as puppies being sold without their mothers, under age, and/or away from their place of birth) are likely the most accessible identifiers of such provenance.

The COVID-19 pandemic precipitated a global surge of puppy acquisitions, commonly referred to as ‘Pandemic Puppies’ (Packer *et al.* 2021, 2023; Siettou 2021; Brand *et al.* 2022, 2024; Burnett *et al.* 2022). Motivated by longer-term welfare concerns for these puppies, the Royal Veterinary College (RVC) launched the ‘Pandemic Puppies Research Programme’ (The Royal Veterinary College 2024) as a longitudinal cohort study in 2020. This cohort study used repeated online questionnaires to explore pre-purchase and purchase decisions of owners and the health and behaviour of both puppies bought before the pandemic (23 March–31 December 2019) and those ‘Pandemic Puppies’ bought during the early phase (23 March–31 December 2020) and mid-phases (23 March–31 December 2021) of the pandemic (Packer *et al.* 2021, 2023). Amongst the 2020-acquired Pandemic Puppy cohort, there was an overall increase in owner purchasing behaviours which obscured the transparency of how and from where their puppies were bred and raised, increasing the risk of acquisition from poor-welfare sources (Packer *et al.* 2021, 2023; Brand *et al.* 2022). For example, owners who purchased a puppy during the 2020 phase of the pandemic were less likely to view their puppy in-person prior to purchase, less likely to see the mother at purchase and more likely to collect their puppy away from their place of birth compared with owners who purchased puppies pre-pandemic in 2019 (Packer *et al.* 2021, 2023; Brand *et al.* 2022). Some of these negative changes persisted and became normalised into 2021 puppy-purchasing practices. For example, the shifts during 2020 towards owners viewing their puppy pre-purchase over video calls or via video recordings/photos rather than in-person, and for collecting their puppy from outside of the seller’s property rather than inside had persisted into 2021 and not returned to pre-pandemic levels (Packer *et al.* 2023). Year-on-year significant rises in the proportion of puppies sold with an international pet passport were also documented between 2019 and 2021, with an increasing number of these puppies being under the minimum legal age for import into the UK at sale (Packer *et al.* 2023). The Pandemic Puppy Research Programme therefore capitalised upon this ‘natural experiment’ to identify associations between indicators of poor acquisition methods that were inflated during this period and a range of health and welfare outcomes in later life for these dogs.

In addition to owner purchasing behaviours and measures of the health of puppies as they aged, owner perceptions relevant to canine health were also explored in this research programme. Owner expectations of veterinary costs are often not in line with reality, with over half (52%) of UK dog owners finding costs of veterinary practice visits greater than expected (Anderson *et al.* 2024). Financial expectations are crucial to animal welfare, influencing whether, when, and what care is sought, and may serve as a measure of preparedness for dog ownership (Anderson *et al.* 2024; Hickey *et al.* 2024).

Based on data collected within the Pandemic Puppy Research Programme, the current study aimed to examine the impact of puppy-purchasing behaviours on later health outcomes of dogs at 21 months of age in a cohort of UK Pandemic Puppies. Specifically, the study aimed to report the frequency of and identify risk factors for:

1. Owner-reported health disorders: The number of owner-reported health disorder groups per dog at 21 months old;
2. Veterinary care: Owner expectations vs realities of veterinary financial costs at 21 months compared to during early puppyhood (while ≤ 7 months of age in 2020).

In addition to risk factors linked to how and from where a puppy was acquired, a wide range of contextual risk factors for health were also explored and/or statistically accounted for, including owner/household demographics, dog demographics, and early-life (puppy) health and veterinary care.

## Materials and methods

### Ethics approval statement and ethical considerations

This study was granted ethical approval from the Social Science Research Ethical Review Board at the Royal Veterinary College (RVC; URN: SR2020-0259). All participants included in the analysis provided informed consent prior to entering the cohort and at each questionnaire time-point. Dogs included in the survey were owned dogs belonging to owners who voluntarily signed up to the Pandemic Puppies project to answer questions concerning their dog’s health and behaviour. Owners were directed to contact their veterinary practice or an animal behaviourist if they had any concerns about their dog’s health or behaviour, respectively, with resources provided to find veterinary surgeons and accredited animal behaviourists if required. Additionally, owners were provided with resources for mental health support from the NHS (National Health Service) and Samaritans due to potential challenges owners may have experienced during the COVID-19 pandemic.

### Survey design and content

A longitudinal online survey design was used to explore the impact of owner purchasing motivations and behaviours and puppies’ early-life experiences on later health outcomes at 21 months of age. As previously described (Brand *et al.* 2024), the Pandemic Puppies Research Programme included an online questionnaire that was disseminated to owners of dogs enrolled in the Pandemic Puppies cohort when their dog reached 21 months of age. This questionnaire included several question sets exploring dog health outcomes, such as mortality, the number and type of owner-reported disorders, preventative healthcare, vaccination status, diet, accessed veterinary care and owner perceptions of the expectations vs realities of veterinary costs. Full information on the questions included in the current analysis are described below, with specific wording of questions relevant to dog health outcomes included in the current study available in File S1 (Supplementary material). The questionnaire also covered other topics that were not considered within the current analysis.

In addition, data regarding owner and dog demographics, and purchase motivations and behaviours were available for the current analysis from the original 2020 questionnaire that had been disseminated soon after the puppies were acquired (Packer *et al.* 2021; Brand *et al.* 2022). Updated dog neuter and insurance status were re-collected during the 21-month questionnaire. If participants had purchased more than one puppy during 23 March and 31 December 2020 they were asked to answer the questionnaire for the youngest puppy and, if the owner had bought littermates, they were asked to answer the questionnaire for the puppy whose name came first alphabetically.

### Participant recruitment

Owners completing the first 2020 Pandemic Puppies questionnaire (Packer *et al.* 2021; Brand *et al.* 2022) who had provided a valid email address and indicated willingness to be contacted regarding further research were sent a unique link to the 21-month questionnaire two weeks prior to their dog turning 21 months of age (639 days). Up to 28 days was allowed for completion. As previously reported (Brand *et al.* 2024), the 21-month questionnaire was hosted using a version of the Vanderbilt University’s Research Electronic Data Capture platform (REDCap) provided by the RVC, a part of the REDCap Consortium (Harris *et al.* 2009, 2019).

### Data cleaning and variable categorisation

Raw data were exported from REDCap into Microsoft Excel® for cleaning prior to data analysis. Dogs were categorised by breed/crossbreed into three groups: purebred; designer crossbred; or crossbred (Brand *et al.* 2022; Burnett *et al.* 2022). Dogs in the purebred group were further categorised according to the Royal Kennel Club (UK) breed grouping where applicable (The Royal Kennel Club 2024). Dog breed was included in analyses as the top 12 most common individual breeds/designer crossbreeds in 21-month survey responses plus ‘other purebred/designer crossbreeds’. Dog breed/crossbreed information was also used to assign a typical adult bodyweight (kg) per dog as previously described (Brand *et al.* 2022) that was categorised as ≤ 10 kg, 10 to < 20 kg, 20 to < 30 kg, 30 to < 40 kg, and ≥ 40 kg.

#### Owner-reported canine health disorder groups

Owners answered three questions relating to their dog’s health between the first survey when dogs were aged ≤ 7 months and the 21-month survey:

- A free-text question regarding new health problem(s) their dogs had been to a veterinary clinic(s) for, and related treatments, herein referred to as ‘veterinary treated disorders’;
- A free-text question regarding any ongoing health problem(s) that still required appointments/treatment/monitoring, herein referred to as ‘ongoing disorders’; and
- A multiple-choice-based question asking whether their dog had shown any of a list of 22 disorders, regardless of whether veterinary care had been sought, with a free-text option allowing the owner to record additional disorders, herein referred to as ‘all disorders’ (File S1; Supplementary material).

Disorder data from each of the three questions were manually mapped to a list of VetCompass disorder groups for further analysis (O’Neill *et al.* 2021). Briefly, disorder groups mapped the original precise diagnosis terms to a general level of diagnostic precision (e.g. inflammatory bowel disease would map to a gastrointestinal disorder group; vomiting, diarrhoea and eating non-food items would map to enteropathy).

The data from the ‘all disorders’ question set above that captured information on all disorders regardless of whether or not owners had sought veterinary advice or not was curated to identify the number of disorder groups dogs had experienced since the time of the 2020 questionnaire. Owners were presented with 22 common health disorders in dogs (e.g. Diarrhoea and/or runny faeces, Runny and/or red eye[s]) plus a free-text box, and asked to tick one of the following answer options ‘No’, ‘Yes, but my dog did not need veterinary advice/to attend a veterinary appointment’, ‘Yes, and my dog did need veterinary advice/to attend a veterinary appointment’, or ‘I’m not sure/I can’t remember’. Both ‘yes’ responses were collapsed to one category for analyses, regardless of whether veterinary advice or care was sought.

Free-text responses were manually cleaned and allocated to the most appropriate disorder group term based on standard VetCompass terms (O’Neill *et al.* 2021). The 22 precise disorders provided in the questions were re-categorised according to disorder groups, and the re-categorised free-text options were combined with these answer options. Grouped level disorders were reported rather than precise level disorders due to these data being owner-reported. Although limited published data indicate owner reports can be reliably validated by veterinary records (Roine *et al.* 2016; Schmid *et al.* 2026), this has not been tested for all disorder, and so caution was exercised to not rely on the accuracy of the reports at a precise level. Duplicates were not summed; only a yes/no for each disorder group was recorded.

For both ‘veterinary treated’ and ‘ongoing disorders’, responses of ‘No’ for the relevant question (‘In your own words, please describe any health problem[s] and related treatments your dog has been to a veterinary clinic[s] for since the last survey [in November/December 2020]’ or ‘Does your dog have any ongoing health problem[s] that still require appointments/treatment/monitoring?’) and ‘No’ in answer to ‘Has your dog attended a veterinary clinic(s) or had a home visit from a veterinary professional for any health problem(s) since the last survey (in November/December 2020)’, were classed as zero disorders. ‘I’m not sure’/‘I can’t remember’ responses to veterinary treated or ongoing disorders, as appropriate, were classed as missing data. ‘Yes’ responses with free-text on veterinary-treated or ongoing disorders, as appropriate, were included in veterinary-treated and ongoing disorder count data. ‘Yes’ responses with free-text which did not include further details on the disorder(s) were classed as ‘Disorder(s) unrecorded’ and these data were not included in disorder prevalence estimates due to missing information and uncertainty on the number and nature of disorders.

For each dog, the number of disorder groups was summed to create a continuous variable (‘veterinary treated’, ‘ongoing’ and ‘all’). The confidence interval estimates for the numbers of disorder groups overall were derived from standard errors based on approximation to a normal distribution for health disorders with ten or more events (Kirkwood & Sterne 2003) or Wilson approximation for disorders with fewer than ten events (Agresti & Coull 1998). The re-categorisation from precise to group level terms of the answer options allowed disorder groups reported since the time of the 2020 questionnaire to be analysed as follows: Total number of disorder groups combined from ‘veterinary treated’, ‘ongoing’ and ‘all’ variables.

#### Number of veterinary clinic visits or home visits from a veterinary professional

Owners were asked whether their dog had attended a veterinary clinic(s) or had a home visit from a veterinary professional for any health problem(s) since the previous questionnaire (in puppyhood aged ≤ 7 months in November/December 2020). The answer options were ‘No’, ‘I can’t remember’ and ‘Yes (please give an approximate total number of visits for health problems since the last questionnaire below as a whole number)’. The ‘No’ responses were treated as zero visits. The number of visits inputted by owners who answered ‘Yes’ were used as a continuous variable to investigate potential associations with the number of owner-reported visits to a veterinary clinic or home visits from a veterinary professional. ‘I can’t remember’ and ambiguous responses were classed as missing data.

#### Total owner-reported veterinary financial costs

Owners were asked to report in a free-text box the approximate total cost (in £) of veterinary care for their dog’s health problem(s) since the previous questionnaire (in November/December 2020). The question specified ‘health problems’, to exclude preventative care, and specified answers were required to also include any money spent on health problems that formed part of an insurance claim, if relevant, and any monies spent on prescription diets rather than routine or preventative care, such as vaccinations, neutering, microchipping, nail clipping, worming, or routine check-ups, for example, which was specified in the question.

#### Owner expectations of veterinary costs

Owners were asked to compare their expectations when they first acquired their dog with the reality of the amount they had spent at veterinary clinic(s) on their dog’s health since the previous questionnaire (in November/December 2020) and report this as ‘Less than I expected’, ‘As I expected’, ‘More than I expected’ or ‘I’m not sure/I can’t remember’ (File S1; Supplementary material). For the purposes of analysis: ‘Less than I expected’ and ‘As I expected’ were combined as ‘As or less than I expected’, with ‘More than I expected’ the outcome of interest from an animal welfare perspective, as has been previously explored (Anderson *et al.* 2024). Missing data and ‘I’m not sure/I can’t remember’ responses were treated as missing data. This new binary outcome variable (‘As or less than I expected’ vs ‘More than I expected’) was used to investigate associations of dog, owner and acquisition-based factors on whether owners had spent more than they expected at veterinary clinics on their dog’s health since the first questionnaire in puppyhood (in November/December 2020).

### Quantitative analysis

Statistical analysis was carried out using IBM SPSS® Statistics v29 (SPSS Inc, Chicago, IL, USA). Demographic data from the 21-month survey (back-coded from the first questionnaire in puppyhood (in November/December 2020) as appropriate) were described. Descriptive statistics (frequency and percentage) were calculated for all categorical variables. The distribution and normality of continuous data were determined by visually inspecting histograms and descriptive statistics calculated (frequency and percentage).

Separate risk-factor analysis modelling was carried out to evaluate each of two outcome variables:

1. Total number of owner-reported disorder groups per dog (continuous variable); and
2. Owner expectations of veterinary costs (binary categorical variable).

The full list of risk factors (n = 39) assessed during univariable analyses for each of the outcomes above is available in File S2 (Supplementary material). These included owner/household demographics, dog demographics, dog early life healthcare, purchase motivations and behaviours, and indicators of potential illegal puppy sale (as previously described; Brand *et al.* 2024). Binary logistic regression modelling was used to evaluate the veterinary cost expectation outcome variable and generalised linear mixed modelling was used to evaluate number of owner-reported disorder groups (all disorders), with the same approach to model-building used across both models. Risk-factor analysis modelling was also conducted for number of veterinary clinics or home visits from a veterinary professional (continuous variable), and total owner-reported veterinary costs (continuous variable) and the results of these models can be found in File S3 (Supplementary material).

Multicollinearity was assessed by examining collinearity diagnostics and standard errors of models for inflated values. Risk factors liberally associated with the outcome variable (*P* ≤ 0.2) in univariable analysis were taken forward to multivariable modelling. Model building used a process of manual backwards stepwise elimination. Following an ‘information theory’ approach (Burnham & Anderson 2002), some variables (owner gender, owner age, first-time ownership, dog neuter status, dog sex, insurance status, dog breed [12 most common breeds in 21-month survey responses]) which were considered *a priori* factors of interest were included in the final model regardless of statistical significance. Model building used the likelihood ratio test to compare between models, with *P* < 0.05 taken as significance. Model fit for each final multivariable model was further assessed by Hosmer-Lemeshow test, adjusted *r*-square values and Akaike Information Criterion, for each model as appropriate. Statistical significance for the final models was set at the 5% level.

Confounding variables were assessed by re-adding variables which were previously dropped and checking for substantial changes (> 20%) to the odds ratio (OR) of variables in the existing final model or any other independent variable following its addition.

## Results

Valid responses were received from n = 1,007 of the 1,742 (57.81%) Pandemic Puppies 2020 participants that were invited to respond. Of these 1,007, n = 9 (0.89%) reported their dog had died under the age of 21 months, of which five were euthanased (55.6%), and four died unassisted (44.4%) (two died following road traffic accidents (50.0%) and two died from other causes (50.0%)). A further n = 13 owners had rehomed their dog under the age of 21 months. The final analysis for the current study therefore included n = 985/1,007 (97.8%) dogs and their owners, that are described from here.

### Dog demographics and acquisition factors

Among the 985 dogs in the current analysis, there was a similar proportion of male and female dogs (male: 52.7%; n = 518/983). Over two-thirds (68.8%) of dogs were purebred (n = 678/985), whilst 26.8% were designer crossbreds (n = 264/985) and 4.4% were crossbreeds (n = 264/985). The 12 most frequent dog breeds/crossbreeds owned by 21-month questionnaire respondents are described in Table 1.

**Table 1.** Most frequent individual dog breeds/crossbreeds in the UK Pandemic Puppies 21-month cohort (n = 985) with % neutered and % male within each breed. Totals for neuter status and sex lower than overall *n* due to missing data

Breed/Crossbreed (Rank)	Frequency% (n)		% Neutered^†^(n)		% Missing Neuter data (n)		% Male(n)		% Missing Sex data (n)	
Labrador Retriever (1)	10.5	(103)	48.5	(50)	4.9	(5)	51.5	(53)	0.0	(0)
Cockapoo (2)	9.1	(90)	75.6	(68)	1.1	(1)	48.9	(44)	1.1	(1)
Cocker Spaniel (3)	7.1	(70)	51.4	(36)	10.0	(7)	55.7	(39)	0.0	(0)
Crossbreed (4)	4.4	(43)	69.8	(30)	9.3	(4)	67.4	(29)	0.0	(0)
Border Collie (5)	3.4	(33)	57.6	(19)	3.0	(1)	33.3	(11)	0.0	(0)
Miniature Smooth-Haired Dachshund (6)	3.1	(31)	12.9	(4)	6.5	(2)	71.0	(22)	0.0	(0)
Border Terrier (7)	3.0	(30)	56.7	(17)	13.3	(4)	56.7	(17)	0.0	(0)
Labradoodle (=8)	2.9	(29)	69.0	(20)	3.4	(1)	37.9	(11)	0.0	(0)
Golden Retriever (=8)	2.9	(29)	41.4	(12)	3.4	(1)	55.2	(16)	0.0	(0)
Whippet (10)	2.3	(23)	60.9	(14)	4.3	(1)	56.5	(13)	4.3	(1)
English Springer Spaniel (11)	2.0	(20)	40.0	(8)	0.0	(0)	70.0	(14)	0.0	(0)
Cavapoo (12)	1.9	(19)	84.2	(16)	5.3	(1)	57.9	(11)	0.0	(0)
Other purebred/designer crossbreed	47.2	(465)	54.0	(251)	5.8	(27)	51.2	(238)	0.0	(0)
	Total n = 985		Total n = 545		Total n = 55		Total n = 518		Total n = 2	

† Neutered by 21 months of age.

Further variables relevant to dog acquisition and demographics that were explored in the statistical modelling are described in Table 2.

**Table 2.** Description of demographic and acquisition-related factors in the UK Pandemic Puppies 21-month cohort (n = 985)

Variable	Category	n	%
Sold without mother present	No	770	78.17
	Yes	213	21.62
	Missing	2	0.20
First-time dog owner	No	604	61.32
	Yes	380	38.58
	Missing	1	0.10
Sold under six weeks of age	No	982	99.70
	Yes	2	0.20
	Missing	1	0.10
Owner saw health test results of mother/father	No	442	44.87
	Yes	508	51.57
	Missing	35	3.55
Owner reported dog had one or more disorders ‘soon after’ acquisition	No	765	77.66
	Yes	220	22.34
	Missing	0	0.00
Owner reported health issues they were concerned about (at time of first questionnaire)	No	924	93.81
	Yes	44	4.47
	Missing	17	1.73
Dog sex	Male	518	52.59
	Female	465	47.21
	Missing	2	0.20
Purebred status	Crossbred	307	31.17
	Purebred	678	68.83
	Missing	0	0.00
Owner age	18–24 years	42	27.61
	25–34 years	188	19.09
	35–44 years	184	18.68
	45–54 years	272	27.61
	55–64 years	184	18.68
	65–74 years	99	10.05
	≥ 75 years	14	1.42
	Missing	2	0.20
Dog insurance status at 21-months	Not insured	117	11.88
	Insured	813	82.54
	Missing	55	5.58
Owner gender	Female	888	90.15
	Male	96	9.75
	Missing	1	0.10
Dog Neutered	No	385	39.09
	Yes	557	56.55
	Missing	43	4.37
Purebred/designer status	Crossbred	43	4.37
	Designer Crossbred	264	26.80
	Purebred	678	68.83
	Missing	0	0.00
Typical adult bodyweight	≤ 10 kg	196	19.90
	10 to < 20 kg	379	38.48
	20 to < 30 kg	214	21.73
	30 to < 40 kg	137	13.91
	≥ 40 kg	17	1.73
	Missing	42	4.26
Breed group	Not KC recognised	315	31.98
	Gundog	289	29.34
	Hound	92	9.34
	Pastoral	66	6.70
	Terrier	91	9.24
	Toy	28	2.84
	Utility	69	7.01
	Working	35	3.55
	Missing	0	0.00
Owner took dog to a vet for any health problem(s) after they brought them home (at the time of the first questionnaire)	No	691	70.15
	Yes	280	28.43
	Missing	14	1.42
Breeder gave worming treatment before owner took puppy home	No	87	8.83
	Yes	888	90.15
	Missing	10	1.02
Sold without microchip details	No	923	93.71
	Yes	15	1.52
	Missing	47	4.77
Purchased breed/crossbreed due to long life-expectancy	No	830	84.26
	Yes	155	15.74
	Missing	0	0.00
Owner paid deposit to secure puppy	No	273	27.72
	Yes	696	70.66
	Missing	16	1.62
Breeder had provided puppy with first vaccinations prior to acquisition	No	306	31.07
	Yes	669	67.92
	Missing	10	1.02
Purchased breed/crossbreed for exercise encouragement	No	625	63.45
	Yes	360	36.55
	Missing	0	0.00

#### Breeding and neutering plans

Neuter information was available for 95.63% of dogs (942/985), with 59.13% of dogs neutered (n = 557/942). Sex-neuter status was available for 940/985 dogs (95.43%). There were 187/385 (48.57%) entire males and 198/385 (51.43%) entire females.

Of the 385 entire dogs at 21 months, n = 294 (76.36%) owners reported that they did not plan to breed from their dog, with 85/385 (22.08%) reporting that they had not bred from their dog yet, but planned to in the future, while n = 2/385 (0.52%) had already bred from their dog.

Of the 385 owners of the entire dogs, 64.16% (n = 247/385) were undecided on whether to neuter their dog, while the remaining n = 138/385 (35.84%) intended to in the future. Of the owners who provided further information regarding the age at which their dog was neutered, 8.81% were neutered under 6 months (n = 48/545), 91.01% (n = 496/545), neutered over 6 months without breeding from them first whilst one owner neutered their dog aged over 6 months after breeding from them first.

#### Microchipping and insurance

Overall, 922/985 owners (93.60%) provided information on whether their dog had been microchipped and the details kept current on a microchip database. Only 1/922 (0.11%) owner reported their dog had not been microchipped by 21 months of age. Of the 921 owners whose dogs were microchipped, 896 (97.29%) reported a microchip database held details of their current name and/or address, whilst 22 (2.39%) had failed to update the microchip to include changes of name/address, and the remaining 3 (0.33%) were unsure whether the microchip details were current. Of the 930 dogs with insurance status reported, 813 (87.42%) were insured for the cost of veterinary advice/treatment at 21 months.

#### Vaccination and preventative parasite control in 21 month old dogs

Of the 929/985 (94.31%) owners who provided information, 869/929 (93.54%) reported their dog had received their initial vaccinations as a puppy and that they planned for them to have an annual booster or had already had an annual booster. Fifty-two dogs (5.60%) had received their initial vaccinations as puppies, but their owners did not plan to continue with an annual booster in the future. The remaining 8/929 owners (0.86%) reported that their dogs had not had their initial vaccinations as a puppy: four (0.43%) owners had actively chosen not to vaccinate their dogs and did not plan to in the future, two (0.22%) owners had not had their dogs vaccinated but planned to in the future and two (0.22%) owners had not had their dogs vaccinated and were unsure whether to vaccinate their dogs in the future. Of the 58/929 (6.24%) owners who did not plan to, or were unsure if they wanted to vaccinate their dog in the future, 20.68% (n = 12/58) had used blood testing/titre testing to assess whether their dog needed to be vaccinated, 41.38% (n = 24/58) planned to use titre testing in the future but had not done so at the time of the questionnaire, 12.07% (n = 7/58) were unsure or had not heard of titre testing, and 25.86% (n = 15/58) were not using or planning to use titre testing in the future.

Of 930/985 (94.42%) owners who provided information on whether their dog received regular flea and/or worming treatment, 881/930 (94.73%) reported that their dog received regular flea and/or worming treatment, with 695/881 (78.89%) of these acquiring these treatments through a veterinary clinic or online via a prescription, while 19.18% (n = 169/881) acquired these treatments at a shop or online without prescription. A minority of owners used natural or homeopathic remedies (3.63%, n = 32/881), while 49/930 (5.27%) had chosen not to use any products regularly to prevent fleas and/or worms.

### Disorder count

#### Disorders for which veterinary care was sought (Veterinary treated disorders)

Of the 985 dogs in the analysis, n = 538 (54.6%) owners reported health problems that their dog had received veterinary care for during the period between the 2020 questionnaire and turning 21 months of age. Twenty-four owners (2.4%) left the question blank or had indicated that they could not remember whether their dog had attended a veterinary clinic for a health problem since the time of the last survey and were treated as missing accordingly, while eighteen owners (1.8%) indicated their dog had attended a veterinary clinic for health problems but did not provide further details and so were classified as ‘disorder(s) unrecorded’. The remaining n = 405 (41.1%) had not taken their dog to a veterinary clinic for a health problem since the time of the last survey, answered ‘none’ or gave details of only routine, prophylactic veterinary visits encompassing vaccinations and neuter surgery.

Excluding missing data and ‘disorder(s) unrecorded’, the median disorder count per dog of these n = 943 dogs was 1 disorder (interquartile range [IQR] 0–2, range 0–8) encompassing 43 grouped disorder categories. The most prevalent disorder group categories were enteropathy (n = 170, prevalence 18.0%, 95% confidence interval [CI]: 15.57–20.48), traumatic injuries (n = 97, prevalence 10.3%, 95% CI: 8.35–12.23), ophthalmological disorders (n = 78, prevalence 8.3%, 95% CI: 6.51–10.03), ear disorders (n = 68, prevalence 7.2%, 95% CI: 5.56–8.86), musculoskeletal disorders (n *=* 64, prevalence 6.8%, 95% CI: 5.18–8.39), and skin disorders (n *=* 64, prevalence 6.8%, 95% CI: 5.18 8.39) (full details in File S4; Supplementary material).

#### Chronic health disorders requiring ongoing veterinary care (Ongoing disorders)

From the n = 985 dogs, n = 947 owners (96.14%) provided subsequent information regarding ongoing health problems requiring monitoring/treatment. Of these owners, n = 855 (90.29%) indicated in closed-ended questions that their dog had no ongoing health problems requiring monitoring/treatment. A total of n = 92 owners left additional free-text regarding ongoing health problems requiring monitoring/treatment. Of these, n = 11 left responses detailing costs, hydrotherapy, or check-ups, but did not give an indication of the specific health problem being managed and were categorised as ‘disorder(s) unrecorded’. The remaining n = 81 responses indicated their dog did have ongoing health problems requiring monitoring/treatment encompassing 25 grouped level disorder terms. Excluding missing data and ‘disorder(s) unrecorded’, the median disorder count was 0 ongoing disorders per dog (IQR 0–0, range 0–3) (n = 936). The most prevalent disorder group categories for ongoing health problems requiring monitoring/treatment were skin disorders (n = 28, prevalence 2.99%, 95% CI: 1.90–4.08), musculoskeletal disorders (n = 13, prevalence 1.39%, 95% CI: 0.64–2.14), behaviour disorders (n = 8, prevalence 0.85%, 95% CI: 0.43–1.68), ear disorders (n = 7, prevalence 0.75%, 95% CI: 0.36–1.54%) and enteropathy (n *=* 4, prevalence 0.43%, 95% CI: 0.17–1.09) (full details in File S4; Supplementary material).

#### Common health disorders regardless of whether or not veterinary advice was sought (all disorders)

From the n = 985 dogs, n = 931 owners (94.52%) answered a separate multiple-choice question that also offered a free-text answer option reporting what disorder(s) their dog had experienced since the previous questionnaire in November/December 2020, regardless of whether they had sought veterinary advice or not. The most prevalent disorder group categories for all health disorders, regardless of whether veterinary advice was sought, were enteropathy (n = 702, prevalence 75.4%, 95% CI: 72.54–78.06), skin disorder (n = 249, prevalence 26.8%, 95% CI: 24.00–29.68), ophthalmological disorders (n = 234, prevalence 25.1%, 95% CI: 22.45–28.02), upper respiratory tract disorders (n = 172, prevalence 18.5%, 95% CI: 16.11–21.10) and ear disorders (n = 164, prevalence 17.6%, 95% CI: 15.30–20.19). Overall, 91.14% (n = 851/931) of owners reported their dog had experienced at least one health problem from this list/free-text answers; the median disorder count per dog was 2 (IQR 1–3, range 0–9) (full details in File S4; Supplementary material).

#### Factors associated with ‘all disorders’ reported between the first questionnaire in puppyhood and reaching 21 months of age

Seventeen of the 39 variables assessed using univariable generalised linear mixed modelling were liberally associated (*P* < 0.2) with the number of disorder groups reported in each dog (see File S5; Supplementary material). The final multivariable model included nine significant variables (Table 3). Five variables were retained in the final model to improve fit, but were not significantly associated with the outcome following manual addition of variables included based on information theory. The marginal pseudo R-squared of the final model was 0.135 meaning the model explained 13.5% of variability in the number of disorder groups (Table 3). Dogs that were sold under six weeks of age, sold without their mother present, owned by a first-time dog owner, where their owner saw health test results of their mother/father, whose owner reported their dog had one or more disorders ‘soon after’ acquisition or reported health issues they were concerned about at time of first ‘puppyhood’ questionnaire, were male or purebred were associated with a higher number of health disorders at 21 months.

**Table 3.** Final multivariable generalised linear mixed model evaluating risk factors association with the number of ‘all disorders’ reported in 21-month old dogs between the first questionnaire in puppyhood (aged ≤ 7 months, in November/December 2020) and dogs aged 21-months (January to August 2022) amongst a cohort of UK Pandemic Puppies acquired < 16 weeks (n = 841 due to missing data across different categories).

Variable	Category	Coefficient	Std. Error	95% CI^*^	*P*-value
Sold under six weeks of age	No			Ref	
	**Yes**	**2.71**	**1.06**	**0.63 – 4.79**	**0.011**
Sold without mother present	No			Ref	
	**Yes**	**0.30**	**0.12**	**0.06 – 0.54**	**0.013**
^†^First-time dog owner	No			Ref	
	**Yes**	**0.35**	**0.12**	**0.12 – 0.58**	**0.003**
Owner saw health test results of mother/father	No			Ref	
	**Yes**	**0.28**	**0.10**	**0.08 – 0.48**	**0.007**
Owner reported dog had one or more disorders ‘soon after’ acquisition	No			Ref	
	**Yes**	**0.32**	**0.13**	**0.0 –0.58**	**0.015**
Owner reported health issues they were concerned about (at time of first questionnaire)	No			Ref	
	**Yes**	**0.69**	**0.25**	**0.21–1.17**	**0.005**
^†^Dog sex	Female			Ref	
	**Male**	**0.20**	**0.10**	**0.00 –0.39**	**0.047**
^†^ ^‡^Purebred status	Crossbred			Ref	
	**Purebred**	**0.11**	**0.11**	**0.04–0.48**	**0.022**
^†^Owner age	18–24 years	0.35	0.27	–0.19–0.88	0.204
	25–34 years	–0.07	0.15	–0.36–0.23	0.658
	35–44 years	–0.24	0.15	–0.53–0.05	0.098
	45–54 years			Ref	**0.049**
	**55–64 years**	**–0.34**	**0.15**	**–0.64 – (–0.05)**	**0.023**
	**65–74 years**	**–0.42**	**0.18**	**–0.78– (–0.06)**	**0.022**
	≥ 75 years	–0.36	0.41	–1.16–0.45	0.382
^†^Dog insurance status at 21-months	Not insured			Ref	
	Insured	0.07	0.15	–0.24–0.37	0.669
^†^Breed	Crossbred			Ref	0.456
	Border Collie	0.10	0.38	–0.64–0.84	0.782
	Border Terrier	0.04	0.39	–0.71–0.80	0.910
	Cavapoo	–0.10	0.43	–0.95–0.75	0.821
	Cockapoo	0.21	0.30	–0.38–0.79	0.487
	Cocker Spaniel	0.34	0.31	–0.27–0.94	0.280
	English Springer Spaniel	0.42	0.41	–0.40 – 1.23	0.314
	Golden Retriever	0.26	0.38	–0.48–1.01	0.488
	Labradoodle	0.18	0.37	–0.55–0.90	0.631
	Labrador Retriever	0.31	0.29	–0.26–0.88	0.283
	Miniature Smooth-Haired Dachshund	–0.14	0.38	–0.88–0.59	0.703
	Other	0.32	0.26	–0.19–0.82	0.217
	Whippet	0.98	0.39	0.21–1.76	0.013
^†^Owner gender	Female			Ref	
	Male	–0.33	0.17	–0.67–0.01	0.056
^†^Dog Neutered	No			Ref	
	Yes	0.04	0.11	–0.17–0.25	0.695
^†^ ^‡^Purebred/designer status	Crossbred			Ref	0.066
	Designer Crossbred	0.125	0.26	–0.39–0.64	0.636
	Purebred	0.368	0.25	–0.13–0.87	0.149
^†^ ^‡^Typical adult bodyweight	≤ 10 kg	–0.22	0.14	–0.49–0.05	0.103
	10 to < 20 kg			Ref	0.067
	20 to < 30 kg	0.12	0.13	–0.14–0.37	0.374
	30 to < 40 kg	0.27	0.15	–0.03–0.57	0.080
	≥ 40 kg	0.13	0.38	–0.60–0.87	0.725
^†^ ^‡^Breed group	Not KC recognised			Ref	0.214
	Gundog	0.36	0.13	0.09–0.62	0.008
	Hound	0.13	0.19	–0.23–0.49	0.482
	Pastoral	0.16	0.22	–0.28–0.60	0.470
	Terrier	0.20	0.20	–0.18–0.59	0.303
	Toy	0.33	0.31	–0.29–0.95	0.291
	Utility	0.30	0.20	–0.10–0.70	0.135
	Working	0.55	0.27	0.02–1.08	0.044
^°^Owner took dog to a vet for any health problem(s) after they brought them home (at the time of the first questionnaire)	No			Ref	
	Yes	0.08	0.12	–0.15–0.32	0.496
^°^Breeder gave worming treatment before owner took puppy home	No			Ref	
	Yes	–0.14	0.19	–0.51–0.22	0.442
^°^Sold without microchip details	No			Ref	
	Yes	0.69	0.56	–0.41–1.79	0.217
^°^Purchased breed/crossbreed due to long life-expectancy	No			Ref	
	Yes	–0.21	0.14	–0.48–0.06	0.121
^°^Owner paid deposit to secure puppy	No			Ref	
	Yes	0.17	0.11	–0.0 –0.40	0.126

† Included as per information theory as a variable of *a priori* interest, regardless of significance.

‡ Dog demographic variables individually used to replace breed (most common 12 breeds at 21-months) in the original model.

° Retained in model for improved model fit.

* 95% confidence interval for the coefficient. Significant results (*P* < 0.05) shown in bold.

### Veterinary visits

Information on whether dogs were registered at a veterinary clinic at 21 months of age was available for 968/985 dogs (98.27%). The majority of dogs 967/968 (99.89%) were registered at a veterinary clinic at 21 months of age, with n = 17 owners not answering this question. The single owner who had not registered for veterinary care stated they only intended to register if their dog became ill. Overall, 564/985 owners (57.26%) reported that their dog had visited a veterinary clinic or had received a home visit from a veterinary professional at least once since their puppyhood questionnaire aged ≤ 7 months, and of these, the median number of visits was 1 (IQR: 2, range: 0–20). Risk factor modelling results are available in File S3 (Supplementary material). Briefly, dogs belonging to experienced dog owners, and whose owners reported they had not taken their dog to the vet for any health problems since they brought them home showed a significant association with a higher number of veterinary visits.

### Veterinary costs and access to veterinary care

The median cost of veterinary care since their questionnaire in puppyhood aged ≤ 7 months for the 985 dogs in the analysis was reported by owners to be £250 (IQR: £550, range: £0–£15,000). For descriptives on ease of veterinary access (e.g. ease owners could get an appointment with their first-choice veterinary clinic or vet, whether owners could accompany their dogs into veterinary consultations) see File S6 (Supplementary material). Risk factor modelling results are available in File S3 (Supplementary material). Briefly, dogs being insured and a typical adult bodyweight of 30 to < 40 kg were significantly associated with higher veterinary costs while owners being aged 75 years old or older was significantly associated with lower veterinary costs.

### Risk factor analysis for owner expectations of veterinary costs at 21 months

Information on the amount owners had spent at veterinary clinic(s) compared to their expectations of veterinary costs when they first acquired their dog were available for 936/985 (95.03%) owners. Six hundred and seven owners (607/936; 64.85%) reported that the amount they spent was as they had expected at acquisition, 220/936 (23.5%) of owners reported that they had spent more than they expected and 98/936 (10.47%) reported that they had spent less than they had expected, while 11/936 owners were not sure or could not remember (1.18%).

Univariable logistic regression identified 18/57 variables were liberally associated (*P* < 0.2) with owners reporting that they had spent more than expected at veterinary clinics on their dog’s health since the 2020 puppyhood questionnaire aged ≤ 7 months. The final multivariable model included seven significant variables (Table 4) and the information theory variables previously described. The Hosmer-Lemeshow test indicated acceptable model fit (*P* = 0.668). Dogs that were sold without a microchip, whose owners saw the health test results of their puppy’s mother/father, whose breeder provided their puppy with their first vaccinations prior to acquisition, whose owner reported health issues they were concerned about at time of first questionnaire, who purchased breed/crossbreed for exercise encouragement, owners of female dogs and owners of dogs who were insured were more likely to have spent more than expected at veterinary clinics on their dog’s health since the 2020 puppyhood questionnaire aged ≤ 7 months.

**Table 4.** Final multivariable binary logistic regression model for owners reporting that they had spent more than they expected at veterinary clinics on their dog’s health since the first questionnaire in puppyhood (in November/December 2020 aged ≤ 7 months) amongst a cohort of UK Pandemic Puppies acquired < 16 weeks (n = 846)

Variable	Category	Odds Ratio	95% CI^*^	*p*-Value
Dog sold without microchip details	No		Ref	
	**Yes**	**5.59**	**1.19–26.37**	**0.030**
Owner saw health test results of mother/father	No		Ref	
	**Yes**	**1.53**	**1.07–2.18**	**0.019**
Breeder had provided puppy with first vaccinations prior to acquisition	No		Ref	
	**Yes**	**1.51**	**1.02–2.25**	**0.041**
Owner reported health issues they were concerned about (at time of first questionnaire)	No		Ref	
	**Yes**	**2.52**	**1.23**–**5.16**	**0.012**
Purchased breed/crossbreed for exercise encouragement	No		Ref	
	**Yes**	**1.52**	**1.07**–**2.14**	**0.018**
^†^Dog sex	Male		Ref	
	**Female**	**0.68**	**0.48**–**0.96**	**0.027**
^†^Dog insurance status at 21-months	Not insured		Ref	
	**Insured**	**2.71**	**1.38–5.35**	**0.004**
^†^Owner age	18–24 years	0.50	0.17–1.47	0.209
	25–34 years	0.73	0.43–1.24	0.250
	35–44 years	1.02	0.62–1.68	0.932
	45–54 years		Ref	0.085
	55–64 years	1.27	0.76–2.11	0.358
	65–74 years	2.07	1.12–3.82	0.020
	≥ 75	0.86	0.17–4.24	0.853
^†^ ^‡^Typical adult bodyweight	≤ 10 kg	0.72	0.45–1.17	0.187
	10 to < 20 kg		Ref	0.456
	20 to < 30 kg	0.93	0.60–1.46	0.764
	30 to < 40 kg	0.99	0.60–1.65	0.979
	≥ 40 kg	0.22	0.03–1.85	0.165
^†^First-time dog owner	No		Ref	
	Yes	1.48	1.00–2.18	0.050
^†^Owner gender	Male		Ref	
	Female	0.74	0.40–1.36	0.331
^†^Neutered	No		Ref	
	Yes	1.08	0.75–1.55	0.689
^†^ ^‡^Purebred status	Crossbred		Ref	
	Purebred	0.91	0.63–1.33	0.637
^†^ ^‡^Purebred designer status	Crossbred		Ref	0.885
	Designer Crossbred	1.07	0.43–2.71	0.881
	Purebred	0.97	0.39–2.40	0.951
^†^Breed	Crossbred		Ref	0.099
	Border Collie	0.78	0.19–3.16	0.726
	Border Terrier	0.52	0.12–2.38	0.403
	Cavapoo	0.54	0.11–2.60	0.445
	Cockapoo	1.06	0.38–2.94	0.916
	Cocker Spaniel	0.45	0.14–1.46	0.186
	English Springer Spaniel	1.03	0.24–4.50	0.971
	Golden Retriever	1.10	0.31–3.88	0.885
	Labradoodle	1.28	0.38–4.33	0.696
	Labrador Retriever	0.77	0.28–2.15	0.619
	Miniature Smooth-Haired Dachshund	0.47	0.11–1.98	0.301
	Other	1.11	0.45–2.74	0.830
	Whippet	3.87	1.10–13.56	0.035
^†^ ^‡^Breed group	Not KC recognised		Ref	0.396
	Gundog	0.71	0.45–1.12	0.140
	Hound	0.99	0.53–1.82	0.961
	Pastoral	0.51	0.21–1.23	0.134
	Terrier	0.96	0.49–1.86	0.899
	Toy	1.67	0.64–4.34	0.291
	Utility	1.27	0.66–2.43	0.476
	Working	1.05	0.41–2.69	0.928

† Included as per information theory as a variable of *a priori* interest, regardless of significance.

‡ Dog demographic variables individually used to replace breed (most common 12 breeds at 21-months) in the original model.

°Retained in model for improved model fit.

* 95% confidence interval. Significant results are emboldened.

## Discussion

This study investigated the longer-term health outcomes of puppies as they reached 21 months of age, identifying risk factors related to their acquisition and early-life experiences. The context of this cohort (puppies acquired during the COVID-19 pandemic) permitted examination of purchase-related risk factors, such as owners not observing their puppy with their mother and illegal (under)age at acquisition, for example, which were naturally inflated during this period, that would otherwise be more challenging to capture in such high numbers.

Our results indicate that the way in which puppies are acquired does have serious longer-term effects on their health, with age at sale and whether a puppy’s mother was present at sale as two key risk factors associated with a higher number of health disorders. Addressing and improving owner behaviour for these two acquisition practices could help to protect the health of future puppies purchased in the UK. Dogs purchased under 6 weeks of age were more likely to have had a higher number of owner-reported health disorder categories by the 21-month time-point than puppies purchased over 6 weeks of age. Puppies advertised for sale under the legal age (8 weeks; UK Government 2013, 2018, 2021a,b) should be seen as a ‘red flag’ to potential purchasers (DEFRA 2020), indicating a higher likelihood of other illegal breeding and sales practices, potentially indicating puppies originate from low-welfare sources. Our findings extend previous research which reported that puppies sold under 8 weeks of age (in the case of that particular study, 6 weeks of age vs 12 weeks of age) were more likely to have short-term negative health consequences including greater weight loss, disease susceptibility, and mortality by six months of age (Slabbert & Rasa 1993). Furthermore, our results indicate that puppies sold without their new owner seeing the puppy’s mother were more likely to experience a higher number of health disorders by 21 months of age compared to puppies whose owners had seen their dog’s mother prior to purchasing their puppy. This is a novel finding, with research to date on puppies being sold without their mother being present at sale having largely been limited to focusing on behavioural outcomes, with negative effects of being sold without a puppy’s mother present on future behaviour (Pierantoni *et al.* 2011; Westgarth *et al.* 2012). These current findings are of high importance and build on previous work by our research team that identified that puppies purchased in 2020 were less likely to be seen with their mother (–10% decline; 75.1% in 2020 vs 85.7% in 2019), and more likely to be purchased at a younger age (7–8 weeks; 67.3%) than puppies purchased in 2019 (in 2019; 52.5%) (Packer *et al.* 2021). Although the proportion of owners who saw their puppy’s mother had returned back to pre-pandemic levels in 2021, > 15% of puppy purchasers still did not engage in this important purchasing behaviour, i.e. were happy to proceed with a sale despite not seeing their puppy’s mother (Packer *et al.* 2023). This is despite owners reporting that a breeder allowing them to see their puppy’s mother was the most desirable breeder characteristic both pre-pandemic (2019), peak-pandemic (2020) and later-pandemic (2021), a desire that remained stable across 2019 to 2020, but concerningly saw a decline in 2021 (Packer *et al.* 2023). This intention-behaviour gap (i.e. the disparity between what people intend to do and what they actually do; Faries 2016) requires targeted human behaviour change interventions to reinforce the importance of not compromising on this purchasing behaviour when making final purchasing decisions. The large proportion of owners who are still buying puppies underage or without seeing their puppy with their mother may suggest that some prospective puppy owners are unaware of the relevant legislation regarding puppy sales, are unable to identify illegal sales indicators (e.g. due to lack of critical analysis of the information presented in puppy adverts, or vital questions to ask puppy sellers), or are aware of the (il)legality of the situation surrounding their puppy’s sale but their desire to purchase a puppy, or that specific puppy overrides their ethical, legal and welfare concerns surrounding the sale (e.g. a wish to ‘save’ their puppy from their sale environment, or an extremely strong desire to acquire a puppy at that specific time). Indeed, previous research has documented that owners commonly acquire dogs in a rapid, unplanned way without hesitation or conducting pre-purchase research, often motivated by the desire to help a vulnerable animal, rapid attachments being built (‘falling in love with the dog’) once encountering an animal in need of a home, and the attractive appearance of dogs in adverts or in person (Holland *et al.* 2021). The population of dogs in the current study were purchased during 2020 where there was a well-documented unprecedented demand for puppies (e.g. 420 potential buyers per puppy advertised in May 2020; Pets4Homes 2020; Siettou 2021) that may have pressurised owners into a scarcity mindset (‘fear of missing out’; Zhang *et al.* 2022), precipitating purchasing puppies from unscrupulous sources in a market where demand greatly exceeded supply. It is clear that further interventions are urgently required in addition to existing efforts (e.g. Defra’s ‘Petfishing’ campaign; DEFRA 2021) to alert the public to illegal sale indicators, stressing the likely negative behavioural and health consequences of purchasing from these sources upon their future dog, which could impact their dog’s welfare, but also their own psychological well-being due to caregiver burden, particularly if disorders are chronic (Spitznagel *et al.* 2017, 2018, 2019, 2021, 2022; Pergande *et al.* 2020; Kuntz *et al.* 2023; Silva *et al.* 2024; Zoanetti *et al.* 2024). Our results provide the evidence to strengthen such campaigns by providing evidence on the negative health outcomes associated with illegal sale indicators, which was previously scarce.

Further to acquisition-based risk factors, we also found that first-time owners were more likely to report a higher number of health disorder groups. A previous study by this research team identified that purchasers of puppies during the pandemic were more likely to be first-time owners than pre-pandemic 2019 puppy purchasers (Packer *et al.* 2021), with two-in-five Pandemic Puppy owners reporting they had no previous experience of owning a dog. First-time dog owners are reported to differ from experienced dog owners in a variety of ways, with these differences largely negative for canine welfare. Lack of experience of dog ownership has been associated with an increased risk of canine behavioural problems in a variety of international studies (Jagoe & Serpell 1996; Kobelt *et al.* 2003; Bennett & Rohlf 2007). First-time owners are also reported to have a reduced bond with their dogs and are more likely to relinquish them (Kidd *et al.* 1992; Scarlett *et al.* 1999; Meyer & Forkman 2014). In the current study population, we have previously reported that first-time dog owners were more likely to report that training their dog at 21 months old was harder than they had expected (Brand *et al.* 2024). It is possible that first-time owners are more likely to seek professional advice when health problems arise or that illness events are more stressful to them and therefore more memorable (and more likely to be reported in questionnaire responses) for first-time owners than experienced owners. The implications of first-time owners reporting higher numbers of health disorder categories could potentially lead to them being unable to cope with caring for their dog leading to relinquishment, especially with the current additional pressures of the cost-of-living crisis placed on many dog owners (Dogs Trust 2022). The association between first-time owners and the high levels of reported health disorder categories in their dogs could be investigated longitudinally to help us support these owners in the future, with specific resources developed to support this vulnerable ownership group.

Several demographic characteristics of dogs and their owners were associated with the number of health disorders reported, which were similar to associations identified in existing published disorder data for UK dogs. For example, male dogs were likely to have a higher number of disorders than female dogs at 21 months of age. This supports previous UK studies where males were reported at higher risk of many common disorders, including otitis externa, osteoarthritis, and seizures (O’Neill *et al.* 2021), and to have a higher median annual disorder count than females (O’Neill *et al.* 2021), although the reasons for these patterns are not clear and require further investigation.

Our results indicated that the majority (91.14%) of dogs at 21 months had experienced one or more health problems (regardless of whether owners sought veterinary advice or not) in the time since the previous questionnaire when the dogs were aged ≤ 7 months. The most common owner-reported disorder groups since the time of the previous questionnaire were enteropathy, skin disorders, eye disorders, upper respiratory tract disorders, and ear disorders. These findings are similar to those reported in this population of puppies while they were under 14 months old (Brand *et al.* 2022), as well as previously reported elsewhere. For example, a study on dogs aged one to three years of age presenting to veterinary clinics in the Republic of Korea reported that the most common disorders in this population were diarrhoea, vomiting, otitis externa, trauma, wounds and ear pruritus (Kim *et al.* 2018). Furthermore, a recent study into the prevalence of disorders of dogs aged under six years of age in a primary veterinary care population in the UK, reported that the most common disorders in these young dogs included infections, allergies, trauma and vomiting/diarrhoea (O’Neill *et al.* 2021). The similarity of the most commonly reported disorders/grouped disorders in this population with those reported previously in dogs overall suggests Pandemic Puppies are subject to the common disorders of the prevailing population structure of dogs at any one time, with little impact from the pandemic on the types of conditions experienced by dogs bought during this time-period over the first 21 months of their lives. However, given many canine inherited disorders do not manifest until later in life, future health profiles may diverge and owners of this cohort of dogs should be encouraged to be vigilant regarding their dog’s health as they age.

The high percentage of owners reporting health disorders in their dogs at 21 months of age (whether or not owners had sought veterinary advice) indicates the need for prospective owners to have realistic expectations of their future dog ownership and the likelihood of their dog experiencing health issues even at a young age, which may require veterinary intervention and thus the financial resources to access this. A mismatch between owner expectations and the reality of dog ownership, such as expectations of health (Salman *et al.* 2000) has been previously reported as a common reason for relinquishment (New Jr *et al.* 2000). High costs of veterinary treatment have long been a common concern of pet owners (Coe *et al.* 2007), and the unexpected cost of veterinary care, whether prophylactic or treatment-based, may lead to some owners feeling that they are unable to continue to keep their dogs (Miller *et al.* 1996). In our study, around one in four owners (23.5%) reported spending more than they had expected on veterinary care for their dog. This figure was lower than reported in other UK research, with Dogs Trust recently finding that 52% of owners felt that the cost of veterinary visits were more than they expected in a large cohort of n = 354,046 dogs (Anderson *et al.* 2024). In a result that may initially appear paradoxical, our study found that owners who had followed a range of positive and recommended practices for puppy acquisition or ownership (e.g. insuring their dog, asking breeders for health tests of their puppy’s parents and sourcing from breeders who had provided their puppy’s first vaccination) were more likely to report spending more than they expected at veterinary clinics. These owners may potentially have been inexperienced and wished to do the ‘right thing’ to maintain their dog’s health, and as well as these positive acquisition/ownership behaviours also followed veterinary advice for positive healthcare behaviours for their dog (e.g. engaging in regular vet checks, preventative healthcare etc) which turned out to be more expensive than they expected, potentially due to the increase in cost-of-living (Beckett 2024; Race 2024). Furthermore, the corporatisation of veterinary care has led to increased veterinary costs in the time since they acquired their dogs as puppies, which owners would not have anticipated (Neate 2024). However, it could alternatively represent that owners following some recommended purchasing/ownership practices may have provided them with a false sense of security that their dog was then somehow ‘protected’ against health issues, e.g. due to buying from a seller they perceived to follow best practice, or that their dog’s parents were free from some inherited disorders they were tested for (O’Neill *et al.* 2020). The widespread misnaming of disorder screening schemes, such as hip, elbow and respiratory function grading as being ‘canine health schemes’, may sell owners on the impression that a dog that passes these tests is therefore a healthy dog and will be guaranteed to produce healthy offspring. However, the reality is that this simply means the dog has passed some tests for one or a few specific disorders that may or may not even be the major health issues for that breed or type of dog (BVA 2024; The Kennel Club 2024), and with the exception of some disorders caused by single gene mutations, screening for most health problems in breeding dogs does not guarantee that puppies of these screened parents will not be affected by them. Many disorder testing schemes do exist for pedigree dog breeds and undoubtedly can play a part in reducing the incidence of these specific disorders (Farrell *et al.* 2015); however, most of the common and serious disorders in dogs have either complex polygenic inheritance, have very low inheritance, or are strongly linked to extreme conformation, and so the currently available formal disorder testing schemes do little to reduce risk for these. Furthermore, disorder testing is likely to only be carried out in a subpopulation that is expected to have a high prevalence of the disorder in question. In contrast to these putatively positive acquisition or ownership behaviours, owners who purchased puppies without a microchip, a non-recommended purchasing practice, and reflecting an illegal sale (since 2016 in England, Wales and Scotland), were more likely to report higher than expected veterinary expenses. Although it may appear paradoxical that both suboptimal and recommended acquisition practices were associated with higher-than-expected veterinary expenditure, these findings likely reflect distinct pathways leading to a similar mismatch between owner expectations and the financial realities of dog ownership. Whereas owners following recommended acquisition and ownership practices may have been well intentioned but overly optimistic about future veterinary care costs, owners purchasing puppies without a microchip acquired their dogs through non-compliant routes and consequently may have encountered unexpected veterinary expenditure due to remedial care requirements and increased underlying health risk associated with this provenance (e.g. due to poor breeding, suboptimal early care).

Ensuring that prospective and current dog owners are prepared for, and have realistic expectations of, potential health issues and associated veterinary costs for their dog is important to avoid undertreatment, relinquishment of dogs due to being unable to meet their care needs, and feelings of guilt in owners who cannot provide expensive but necessary care for their pets (Brockman *et al.* 2008). This is particularly pertinent at present, with veterinary care costs increasing (Lovett 2024; Webb 2024) in tandem with the ongoing cost-of-living crisis in the UK (Beckett 2024; Race 2024). Counselling prospective owners on how to ensure affordability of veterinary costs, including the impact of their choice of dog breed on likely veterinary care costs, is increasingly important in the current financial and veterinary care climate. Encouraging acquisition of breeds with high innate health (ability to live and function without health issues related to extreme conformation; Brachycephalic Working Group 2022), acquired from breeders adhering to legal standards for breeding and selling of puppies, is likely to go some way towards reducing risks of future health problems and associated financial burden for owners.

The finding that dogs belonging to experienced owners, and whose owners reported that they had not taken their dog to a vet for any health problems since acquisition, were significantly associated with a higher number of veterinary visits may initially appear counterintuitive. However, this result likely reflects differences in care-seeking behaviour by experienced dog owners rather than increased morbidity, and engagement with routine and preventive veterinary care rather than problem-driven consultations. Evidence suggests that regular engagement with veterinary teams is associated with more welfare-appropriate decision-making by owners (Merle & Küper 2021; Farrow *et al.* 2026). As a result, dogs may accumulate a higher number of veterinary visits despite the absence of owner-reported illness or injury and indeed may be a protective factor rather than a marker of poor health.

### Study limitations

This study has a number of limitations that were mitigated where possible. The number of owner-reported disorder categories in the study may have been confounded by the atypical nature of the study population. Indeed, the owners answering this questionnaire had already completed a questionnaire with the current research team while their dogs were puppies (< 7 months), and thus may represent a more committed, vigilant owner population, particularly attentive to their dogs’ health. Conversely, this count may underrepresent the extent of ill health experienced in this cohort, as it does not account for the number of precise disorders experienced within a disorder category (e.g. more than one type of skin disorder), nor disorder frequency, severity or duration. As reported previously by the research team, this study population has a disproportionately low number of brachycephalic dogs represented (O’Neill *et al.* 2023; Brand *et al.* 2024) compared to the general UK canine population, as also observed in other longitudinal cohort studies of dogs (Murray *et al.* 2021). This may mean that the overall disease burden in this population was lower, in addition to lower than expected levels of disorders commonly seen in brachycephalic breeds, such as ophthalmic, skin and respiratory disorders (O’Neill *et al.* 2018, 2020).

Disorders/disorder groups are referred to as ‘owner-reported’ disorders in this paper as the authors could not be certain whether disorders were diagnosed by a veterinary professional or were observed and categorised by the owners themselves. This was particularly true where owners identified that their dog had a disorder that did not require a veterinary appointment, meaning no veterinary diagnosis may have occurred; however, this approach allowed for large-scale data collection without the additional challenges of verifying owner reports by requesting that veterinary records were provided, which could have reduced retention levels which were otherwise high, and higher than other studies requesting these data (Murray *et al.* 2021). Similarly, for cost estimates, the values were also referred to as owner-reported as the owners were asked to estimate the cost of veterinary care so the values may not have necessarily represented the true cost of veterinary care since the 2020 questionnaire; however, large-scale databases of veterinary care, such as VetCompass do not collect financial information, and thus suitable alternatives were not available at the time of the study. Reported values for disorders and costs are therefore potentially inaccurate depending on owner recall, albeit this was over a relatively short time-period.

## Animal welfare implications and conclusion

Our results suggest that longer-term canine health outcomes are linked to how and from where a puppy is acquired and that greater purchasing prudence is needed by prospective owners to avoid supporting poor-welfare sources and avoid future health problems in their dog. The disease burden of young adult dogs was negatively influenced by acquisition-based factors, with puppies sold under six weeks of age, or sold without the owners seeing their puppies’ mother – both now illegal in England, Scotland and Wales – associated with a higher number of owner-reported disorder groups at 21 months old. Greater enforcement of this legislation, greater awareness of this legislation by prospective buyers, and human behaviour change interventions to improve the intention-behaviour gap observed here (where owners were previously motivated to follow these practices but did not) are needed to protect canine welfare. Finally, one in four owners felt veterinary care costs for their dog were higher than expected even at this relatively young age of 21 months. More effective education is needed to manage owner expectations prior to acquisition, to reduce expectation-mismatch related relinquishments and undertreatment, particularly in light of rising veterinary costs and the cost-of-living crisis.

## Supporting information

10.1017/awf.2026.10077.sm001 Dale et al. supplementary material Dale et al. supplementary material

## Supplementary material

The supplementary material for this article can be found at http://doi.org/10.1017/awf.2026.10077.

## References

[r1] Agresti A, and Coull BA 1998 Approximate is better than “exact” for interval estimation of binomial proportions. The American Statistician 52: 119–126. 10.2307/2685469

[r2] Anderson KL, Holland KE, Casey RA, Cooper B, and Christley RM 2024 Owner expectations and surprises of dog ownership experiences in the United Kingdom. Frontiers in Veterinary Science 11. 10.3389/fvets.2024.1331793 PMC10880448 38384957

[r3] Beckett A 2024 *The cost of living crisis has made the UK a poorer, more anxious nation – and worse is yet to come.* The Guardian. https://www.theguardian.com/commentisfree/2024/apr/26/cost-of-living-crisis-uk-prices (accessed 16 February 2026).

[r4] Belshaw Z, Brand CL, O’Neill DG and Packer RMA 2025a More than just one man and his dog: The many impacts of puppy acquisition on the mental health of families including children in the UK. PLoS One 20: e0331179. 10.1371/journal.pone.0331179 40961036 PMC12443288

[r5] Belshaw Z and Packer RMA 2025 Knowledge of UK residents about importing puppies from EU Countries. Animals 15: 2193. 10.3390/ani15152193 40804982 PMC12345551

[r6] Belshaw Z, Youens E, Lord M and Packer RMA 2025b “A dog brings benefits no matter where it’s from”: UK residents’ Understanding of the benefits and risks of importing puppies from Romania to the UK. Animals 15: 2192. 10.3390/ani15152192 40804983 PMC12345484

[r7] Bennett PC and Rohlf VI 2007 Owner-companion dog interactions: Relationships between demographic variables, potentially problematic behaviours, training engagement and shared activities. Applied Animal Behaviour Science 102: 65–84. 10.1016/j.applanim.2006.03.009

[r8] Brachycephalic Working Group 2022 *Innate health in dogs.* https://www.ukbwg.org.uk/wp-content/uploads/2022/05/220512-BWG-Innate-health-in-dog-populations.pdf (accessed 16 February 2026).

[r9] Brand CL, O’Neill DG, Belshaw Z, Dale FC, Merritt BL, Clover KN, Tay M-XM, CL and Packer RMA 2024 Impacts of puppy early life experiences, puppy-purchasing practices, and owner characteristics on owner-reported problem behaviours in a UK pandemic puppies cohort at 21 months of age. Animals 14: 336. 10.3390/ani14020336 38275796 PMC10812580

[r10] Brand CL, O’Neill DG, Belshaw Z, Pegram CL, Stevens KB and Packer RMA 2022 Pandemic puppies: Demographic characteristics, health and early life experiences of puppies acquired during the 2020 phase of the COVID-19 pandemic in the UK. Animals 12: 629. 10.3390/ani12050629 35268198 PMC8909199

[r11] Brockman BK, Taylor VA and Brockman CM 2008 The price of unconditional love: Consumer decision making for high-dollar veterinary care. Journal of Business Research 61: 397–405. 10.1016/j.jbusres.2006.09.033

[r12] Burnett E, Brand CL, O’Neill DG, Pegram CL, Belshaw Z, Stevens KB and Packer RMA 2022 How much is that doodle in the window? Exploring motivations and behaviours of UK owners acquiring designer crossbreed dogs (2019-2020). Canine Medicine and Genetics 9: 8. 10.1186/s40575-022-00120-x 35610665 PMC9127489

[r13] Burnham K and Anderson DR 2002 Model Selection and Multimodel Inference: A Practical Information-Theoretic Approach. Springer: New York, NY, USA.

[r14] BVA 2024 *Hip scheme.* British Veterinary Association. https://www.bva.co.uk/canine-health-schemes/hip-scheme/ (accessed 16 February 2026).

[r15] Casey RA, Loftus B, Bolster C, Richards GJ and Blackwell EJ 2014 Human directed aggression in domestic dogs (*Canis familiaris*): Occurrence in different contexts and risk factors. Applied Animal Behaviour Science 152: 52–63. 10.1016/j.applanim.2013.12.003

[r16] Coe JB, Adams CL and Bonnett BN 2007 A focus group study of veterinarians’ and pet owners’ perceptions of the monetary aspects of veterinary care. Journal of American Veterinary Medical Association 231: 1510–1518. 10.2460/javma.231.10.1510 18020992

[r17] Costa AG, Nielsen T, Christley R and Hazel S 2023 The good, the bad, the helpful: Qualitative exploration of the Australian puppy-raising experience through longitudinal surveys. Anthrozoös 36: 869–890. 10.1080/08927936.2023.2238435

[r18] DEFRA 2020 *Lucy’s Law spells the beginning of the end for puppy farming.* Department for Environment, Food & Rural Affairs, UK Government. https://www.gov.uk/government/news/lucys-law-spells-the-beginning-of-the-end-for-puppy-farming (accessed 16 February 2026).

[r19] DEFRA 2021 Petfished: Who’s the person behind the pet? HM Government: London, UK.

[r20] Dendoncker P-A, Moons C, Sarrazin S, Diederich C, Thiry E, de Keuster T and Dewulf J 2018 Biosecurity and management practices in different dog breeding systems have considerable margin for improvements. Veterinary Record 183: 381. 10.1136/vr.104996 30045997

[r21] Dogs Trust 2022 *Cost of living crises hits dog owners across the UK.* https://www.dogstrust.org.uk/about-us/what-we-do/stories/letter-to-chancellor (accessed 16 February 2026).

[r22] Faries MD 2016 Why we don’t “just do it”: Understanding the intention-behavior gap in lifestyle medicine. American Journal of Lifestyle Medicine 10: 322–329. 10.1177/1559827616638017 30202289 PMC6125069

[r23] Farrell LL, Schoenebeck JJ, Wiener P, Clements DN and Summers KM 2015 The challenges of pedigree dog health: approaches to combating inherited disease. Canine Genetics and Epidemiology 2: 3. 10.1186/s40575-015-0014-9 26401331 PMC4579364

[r24] Farrow M, O’Neill DG and Packer RMA 2026 To see or not to see the vet: A vignette-based study of decision-making by UK dog owners regarding seeking veterinary care for commonly presenting conditions. PLoS One 21: e0339723. 10.1371/journal.pone.0339723 41544060 PMC12810856

[r25] Harris PA, Taylor R, Minor BL, Elliott V, Fernandez M, O’Neal L, McLeod L, Delacqua G, Delacqua F, Kirby J, Duda SN and Consortium RE 2019 The REDCap consortium: Building an international community of software platform partners. Journal of Biomedical Information 95: 103208. 10.1016/j.jbi.2019.103208 PMC7254481 31078660

[r26] Harris PA, Taylor R, Thielke R, Payne J, Gonzalez N and Conde JG 2009 Research electronic data capture (REDCap): a metadata-driven methodology and workflow process for providing translational research informatics support. Journal of Biomedical Information 42: 377–381. 10.1016/j.jbi.2008.08.010 PMC2700030 18929686

[r27] Hickey K, Smith D and Shields E 2024 Qualitative research on pet owners’ experiences of buying veterinary services in the UK. https://assets.publishing.service.gov.uk/media/65eedd9d62ff4898bf87b261/Qualitative_Research_on_Pet_Owners__Experiences_of_Buying_Veterinary_Services_in_the_UK.pdf (accessed 16 February 2026).

[r28] Holland KE 2019 Acquiring a pet dog: A review of factors affecting the decision-making of prospective dog owners. Animals 9: 124. 10.3390/ani9040124 30925784 PMC6523466

[r29] Holland KE, Mead R, Casey RA, Upjohn MM and Christley RM 2021 “Don’t bring me a dog… I’ll just keep It”: Understanding unplanned dog acquisitions amongst a sample of dog owners attending canine health and welfare community events in the United Kingdom. Animals (Basel) 11. 10.3390/ani11030605 PMC7996527 33668882

[r30] Jagoe A and Serpell J 1996 Owner characteristics and interactions and the prevalence of canine behaviour problems. Applied Animal Behaviour Science 47: 31–42. 10.1016/0168-1591(95)01008-4

[r31] Jagoe JA 1994 Behaviour problems in the domestic dog: a retrospective and prospective study to identify factors influencing their development. University of Cambridge, UK. 10.17863/CAM.31066

[r32] Kidd AH, Kidd RM and George CC 1992 Successful and unsuccessful PET adoptions. Psychological Reports 70: 547–561. 10.2466/PR0.70.2.547-561

[r33] Kim E, Choe C, Yoo JG, Oh S-I, Jung Y, Cho A, Kim S and Do YJ 2018 Major medical causes by breed and life stage for dogs presented at veterinary clinics in the Republic of Korea: a survey of electronic medical records. PeerJ 6: e5161. 10.7717/peerj.5161 30013835 PMC6035722

[r34] Kinsman RH, Casey RA, Knowles TG, Tasker S, Lord MS, Da Costa REP, Woodward JL and Murray JK 2020 Puppy acquisition: factors associated with acquiring a puppy under eight weeks of age and without viewing the mother. Veterinary Record 187: 112. 10.1136/vr.105789 32764003 PMC7456714

[r35] Kirkwood BR and Sterne JAC 2003 Essential Medical Statistics. Wiley-Blackwell Publishing: Oxford, UK.

[r36] Kobelt AJ, Hemsworth PH, Barnett JL and Coleman GJ 2003 A survey of dog ownership in suburban Australia—conditions and behaviour problems. Applied Animal Behaviour Science 82: 137–148. 10.1016/S0168-1591(03)00062-5

[r37] Kuhl CA, Dean R, Quarmby C and Lea RG 2022 Information sourcing by dog owners in the UK: Resource selection and perceptions of knowledge. Veterinary Record 190: e1081. 10.1002/vetr.1081 34741470

[r38] Kuntz K, Ballantyne KC, Cousins E and Spitznagel MB 2023 Assessment of caregiver burden in owners of dogs with behavioral problems and factors related to its presence. Journal of Veterinary Behavior 64-65: 41–46. 10.1016/j.jveb.2023.05.006

[r39] Lovett D 2024 Veterinary services prices. *Which?* https://www.which.co.uk/policy-and-insight/article/veterinary-services-prices-aDx9O1o7aIdF (accessed 16 February 2026).

[r40] Maher J, and Wyatt T 2021 European illegal puppy trade and organised crime. Trends in Organized Crime 24: 506–525. 10.1007/s12117-021-09429-8 34456550 PMC8382934

[r41] McMillan FD 2017 Behavioral and psychological outcomes for dogs sold as puppies through pet stores and/or born in commercial breeding establishments: Current knowledge and putative causes. Journal of Veterinary Behavior 19: 14–26. 10.1016/j.jveb.2017.01.001

[r42] McMillan FD, Serpell JA, Duffy DL, Masaoud E and Dohoo IR 2013 Differences in behavioral characteristics between dogs obtained as puppies from pet stores and those obtained from noncommercial breeders. Journal of the American Veterinary Medical Association 242: 1359–1363. 10.2460/javma.242.10.1359 23634679

[r43] Merle R and Küper AM 2021 Attitude of veterinarians toward self-informed animal owners affects shared decisSon making. Frontiers in Veterinary science 8. 10.3389/fvets.2021.692452 PMC8564114 34746272

[r44] Meyer I and Forkman B 2014 Dog and owner characteristics affecting the dog–owner relationship. Journal of Veterinary Behavior 9: 143–150. 10.1016/j.jveb.2014.03.002

[r45] Miller DD, Staats SR, Partlo C and Rada K 1996 Factors associated with the decision to surrender a pet to an animal shelter. Journal of the American Veterinary Medical Association 209: 738–742. 10.2460/javma.1996.209.04.738 8756871

[r46] Murray JK, Kinsman RH, Lord MS, Da Costa REP, Woodward JL, Owczarczak-Garstecka SC, Tasker S, Knowles TG and Casey RA 2021 ’Generation Pup’ – protocol for a longitudinal study of dog behaviour and health. BMC Veterinary Research 17: 1. 10.1186/s12917-020-02730-8 33397375 PMC7781182

[r47] Naturewatch 2025 *Licensed dog breeding in the UK and Ireland 2025.* https://naturewatch.org/wp-content/uploads/2025/04/Licensed-dog-breeding-in-the-UK-and-Ireland-2025-by-Naturewatch-Foundation.pdf (accessed 16 February 2026).

[r48] Neate R 2024 ‘The vet presented it as: if you care, you pay’: who really profits from poorly pets? *The Guardian.* https://www.theguardian.com/lifeandstyle/2024/apr/06/vet-who-really-profits-from-poorly-pets (accessed 16 February 2026).

[r49] New Jr JC, Salman MD, King M, Scarlett JM, Kass PH, and Hutchison JM 2000 Characteristics of shelter-relinquished animals and their owners compared with animals and their owners in US pet-owning households. Journal of Applied Animal Welfare Science 3: 179–201. 10.1207/S15327604JAWS0303_1

[r50] O’Neill DG, Baral L, Church DB, Brodbelt DC and Packer RMA 2018 Demography and disorders of the French Bulldog population under primary veterinary care in the UK in 2013. Canine Genetics and Epidemiology 5: 3. 10.1186/s40575-018-0057-9 29750111 PMC5932866

[r51] O’Neill DG, James H, Brodbelt DC, Church DB and Pegram C 2021 Prevalence of commonly diagnosed disorders in UK dogs under primary veterinary care: results and applications. BMC Veterinary Research 17: 69. 10.1186/s12917-021-02775-3 33593363 PMC7888168

[r52] O’Neill DG, McMillan KM, Church DB and Brodbelt DC 2023 Dog breeds and conformations in the UK in 2019: VetCompass canine demography and some consequent welfare implications. PLoS One 18: e0288081. 10.1371/journal.pone.0288081 37494312 PMC10370710

[r53] O’Neill DG, Pegram C, Crocker P, Brodbelt DC, Church DB and Packer RMA 2020 Unravelling the health status of brachycephalic dogs in the UK using multivariable analysis. Scientific Reports 10: 17251. 10.1038/s41598-020-73088-y 33057051 PMC7560694

[r54] Packer RMA, Brand CL, Belshaw Z, Pegram CL, Dale F, Stevens KB and O’Neill DG 2023 Is UK puppy purchasing suffering a long COVID effect? Ongoing negative impacts of the COVID-19 pandemic upon puppy purchase motivations and behaviours in 2021. Animals 13: 2186. 10.3390/ani13132186 37443983 PMC10339900

[r55] Packer RMA, Brand CL, Belshaw Z, Pegram CL, Stevens KB and O’Neill DG 2021 Pandemic puppies: Characterising motivations and behaviours of UK owners who purchased puppies during the 2020 COVID-19 pandemic. Animals 11. 10.3390/ani11092500 PMC8468924 34573466

[r56] Packer RMA, Murphy D and Farnworth MJ 2017 Purchasing popular purebreds: investigating the influence of breed-type on the pre-purchase motivations and behaviour of dog owners. Animal Welfare 26: 191–201. 10.7120/09627286.26.2.191

[r57] PDSA 2024a *Could you spot a puppy farm?* https://www.pdsa.org.uk/pet-help-and-advice/looking-after-your-pet/puppies-dogs/could-you-spot-a-puppy-farm (accessed 16 February 2026).

[r58] PDSA 2024b *PDSA Animal Wellbeing Report 2024.* https://www.pdsa.org.uk/media/14944/pdsa_paw-report-2024.pdf (accessed 16 February 2026).

[r59] Pergande AE, Belshaw Z, Volk HA and Packer RMA 2020 “We have a ticking time bomb”: a qualitative exploration of the impact of canine epilepsy on dog owners living in England. BMC Veterinary Research 16: 443. 10.1186/s12917-020-02669-w 33187534 PMC7666515

[r60] Pets4Homes 2020 *The State of the Pet Industry No.* 1 *- The Impact of COVID-19 on the UK Pet Landscape.* https://www.pets4homes.co.uk/pet-advice/pandemic-pets-how-covid-19-affected-pet-sales-and-pricing-in-2020.html (accessed 16 February 2026).

[r61] Pierantoni L, Albertini M, and Pirrone F 2011 Prevalence of owner-reported behaviours in dogs separated from the litter at two different ages. Veterinary Record 169: 468. 10.1136/vr.d4967 21865608

[r62] Pirrone F, Pierantoni L, Pastorino GQ and Albertini M 2016 Owner-reported aggressive behavior towards familiar people may be a more prominent occurrence in pet shop-traded dogs. Journal of Veterinary Behavior 11: 13–17. 10.1016/j.jveb.2015.11.007

[r63] Race M 2024 *More than 7 million adults still struggling to pay bills, survey finds.* BBC. https://www.bbc.co.uk/news/business-68765769 (accessed 16 February 2026).

[r64] Roine J, Uusitalo L and Hielm-Björkman A 2016 Validating and reliability testing the descriptive data and three different disease diagnoses of the internet-based DOGRISK questionnaire. BMC Veterinary Research 12: 30. 10.1186/s12917-016-0658-z 26897627 PMC4761135

[r65] RSPCA Australia 2024 *What is a puppy farm?* RSPCA Knowledgebase. https://kb.rspca.org.au/categories/companion-animals/choosing-a-pet/what-is-a-puppy-farm (accessed 16 February 2026).

[r66] Salman MD, Hutchison J, Ruch-Gallie R, Kogan L, New JC, Kass PH and Scarlett JM 2000 Behavioral reasons for relinquishment of dogs and cats to 12 shelters. Journal of Applied Animal Welfare Science 3: 93–106. 10.1207/S15327604JAWS0302_2

[r67] Scarlett JM, Salman MD, New JJG and Kass PH 1999 Reasons for relinquishment of companion animals in US animal shelters: Selected health and personal issues. Journal of Applied Animal Welfare Science 2: 41–57. 10.1207/s15327604jaws0201_4 16363961

[r89] Schmid SM, Sexton CL, Yoerger A, Kauffman M, McClelland RL, Consortium DAP, Creevy KE and Ruple A 2026 Accuracy of owner-reported diagnoses for dogs enrolled in the Dog Aging Project as compared to veterinary electronic medical records. PLOS One. 10.1371/journal.pone.0342427 PMC12959718 41779687

[r68] Siettou C 2021 Societal interest in puppies and the Covid-19 pandemic: A google trends analysis. Preventive Veterinary Medicine 196: 105496. 10.1016/j.prevetmed.2021.105496 34555632

[r69] Silva PT, Coura FM and Costa-Val AP 2024 Caregiver burden in small animal clinics: a comparative analysis of dermatological and oncological cases. Animals 14: 276. 10.3390/ani14020276 38254445 PMC10812608

[r70] Slabbert J, and Rasa O 1993 The effect of early separation from the mother on pups in bonding to humans and pup health. Journal of the South African Veterinary Association 64: 4–8. 7802733

[r71] Spitznagel M, Jacobson D, Cox M and Carlson M 2018 Predicting caregiver burden in general veterinary clients: contribution of companion animal clinical signs and problem behaviors. The Veterinary Journal 236: 23–30. 10.1016/j.tvjl.2018.04.007 29871745

[r72] Spitznagel MB, Cox MD, Jacobson DM, Albers AL and Carlson MD 2019 Assessment of caregiver burden and associations with psychosocial function, veterinary service use, and factors related to treatment plan adherence among owners of dogs and cats. Journal of American Veterinary Medical Association 254: 124–132. 10.2460/javma.254.1.124 30668290

[r73] Spitznagel MB, Hillier A, Gober M and Carlson MD 2021 Treatment complexity and caregiver burden are linked in owners of dogs with allergic/atopic dermatitis. Veterinary Dermatology 32: 192–e150. 10.1111/vde.12938 33554382 PMC8048808

[r74] Spitznagel MB, Jacobson DM, Cox MD and Carlson MD 2017 Caregiver burden in owners of a sick companion animal: a cross-sectional observational study. Veterinary Record 181: 321. 10.1136/vr.104295 28870976

[r75] Spitznagel MB, Patrick K, Hillier A, Gober M, and Carlson MD 2022 Caregiver burden, treatment complexity, and the veterinarian–client relationship in owners of dog with skin disease. Veterinary Dermatology 33: 208–213. 10.1111/vde.13065 35293042 PMC9311805

[r76] The Kennel Club 2024 *Respiratory Function Grading Scheme.* https://www.royalkennelclub.com/health-and-dog-care/health-dog-care/health/getting-started-with-health-testing-and-screening/respiratory-function-grading-scheme/ (accessed 16 February 2026).

[r77] The Royal Kennel Club 2024 *Breeds A to Z.* https://www.royalkennelclub.com/search/breeds-a-to-z/ (accessed 16 February 2026).

[r78] The Royal Veterinary College 2024 *RVC Pandemic Puppies Research Programme.* https://www.rvc.ac.uk/vetcompass/research-projects-and-opportunities/projects/rvc-pandemic-puppies-survey (accessed 16 February 2026).

[r79] UK Government 2013 *The Welfare of Animals (Dog Breeding Establishments and Miscellaneous Amendments) Regulations (Northern Ireland)* 2013. https://www.legislation.gov.uk/nisr/2013/43/ (accessed 16 February 2026).

[r80] UK Government 2018 *The Animal Welfare (Licensing of Activities Involving Animals) (England) Regulations 2018.* https://www.legislation.gov.uk/uksi/2018/486 (accessed 16 February 2026).

[r81] UK Government 2019 *The Animal Welfare (Licensing of Activities Involving Animals) (England) (Amendment) Regulations 2019.* https://statutoryinstruments.parliament.uk/instrument/tir8HHhR (accessed 16 February 2026).

[r82] UK Government 2021a *The Animal Welfare (Licensing of Activities Involving Animals) (Scotland) Regulations 2021.* https://www.legislation.gov.uk/ssi/2021/84 (accessed 16 February 2026).

[r83] UK Government 2021b *The Animal Welfare (Licensing of Activities Involving Animals) (Wales) Regulations 2021.* https://www.legislation.gov.uk/wsi/2021/416 (accessed 16 February 2026).

[r84] Wauthier LM, Scottish Society for the Prevention of Cruelty to Animals and Williams JM 2018 Using the mini C-BARQ to investigate the effects of puppy farming on dog behaviour. Applied Animal Behaviour Science 206: 75–86. 10.1016/j.applanim.2018.05.024

[r85] Webb A 2024 Analyst warns rising costs likely to be ‘unsustainable.’ *Vet Times.* https://www.vettimes.com/news/business/finance/analyst-warns-rising-costs-likely-to-be-unsustainable (accessed 16 February 2026).

[r86] Westgarth C, Reevell K, and Barclay R 2012 Association between prospective owner viewing of the parents of a puppy and later referral for behavioural problems. Veterinary Record 170: 517. 10.1136/vr.100138 22562104

[r87] Zhang J, Jiang N, Turner JJ and Pahlevan-Sharif S 2022 The impact of scarcity on consumers’ impulse buying based on the S-O-R Theory. Frontiers in Psychology 13. 10.3389/fpsyg.2022.792419 PMC9231522 35756291

[r88] Zoanetti J, Nielsen TD and Hazel S 2024 The potential negative impacts of pet guardianship on the guardian, a scoping review. Discover Animals 1: 11. 10.1007/s44338-024-00014-1

