# Supplementary material for: Sold a pup? Impact of purchasing practices, owner and dog demographics, and puppy early-life experiences on later canine health outcomes in the UK
Source: Anim Welf. 2026 Apr 1;35:e18. doi: 10.1017/awf.2026.10077 (PMC13100921; doi:10.1017/awf.2026.10077)
Supplement: Dale et al. supplementary material [file S0962728626100773sup001.pdf]

Sold a pup? Impact of purchasing practices, owner and dog demographics, and puppy early life experiences on later canine health outcomes in the UK: Supplementary material

Fiona C Dalehttps://orcid.org/0009-0006-3948-7938[1], Dan G O'Neill[1], Claire L Brandhttps://orcid.org/0000-0001-9670-0324[2], Zoe Belshaw[3], Bree L Merritt[2], Camilla L Pegram[1] and Rowena MA Packerhttps://orcid.org/0000-0002-9988-9828[2]

[1] Department of Pathobiology and Population Sciences, The Royal Veterinary College, Hatfield, Herts AL9 7TA, UK

[2] Department of Clinical Science and Services, The Royal Veterinary College, Hatfield, Herts AL9 7TA, UK

[3] EviVet Evidence-based Veterinary Consultancy, Nottingham, UK

Author for correspondence: Rowena MA Packer, email: rpacker@rvc.ac.uk

File S1

Table S1. Question and answer options presented to owners between the first questionnaire in puppyhood (November/December 2020 aged ≤ 7 months) and turning 21 months of age (between January to August 2022) in a cohort of UK Pandemic Puppies acquired < 16 weeks of age, used for dog health outcomes (owner-reported canine health disorder groups; owner expectations of veterinary costs) The answer options listed represent all the choices available to respondents.

Outcome	Question	Listed Disorders	Available respondent choices
Owner-reported canine health disorder groups	Has your dog shown any of the following since the last survey (in November/December 2020), regardless of whether you sought veterinary advice or not? (all disorders)	• Runny and/or red eye(s)<br>• Diarrhoea and/or runny faeces<br>• Vomiting (being sick)<br>• Worms in faeces<br>• Fleas/other parasites visible in fur/on skin<br>• Hair loss<br>• Wounds/sore areas of skin<br>• Frequent itching/licking<br>• Coughing<br>• Sore or infected ear(s)<br>• Problems with their anal glands<br>• Dental problems<br>• Lameness<br>• Overgrown nails<br>• Being overweight<br>• Losing weight<br>• Arthritis<br>• Lumps	No<br>Yes, but my dog did not need veterinary advice/to attend a veterinary appointment<br>Yes, and my dog did need veterinary advice/to attend a veterinary appointment<br>I’m not sure/I can’t remember

		• Funny turns (suspected/confirmed to be fits/seizures)<br>• Urinary incontinence (leaking urine)<br>• Breathing problems<br>• Eating non-food items (e.g., cloth, plastic, stones, faeces)<br>• Other (please specify below)	
	Does your dog have any ongoing health problem(s) that still require appointments/treatment/monitoring? (ongoing disorders)	N/a	No<br>Yes (please describe below)<br>I'm not sure
	In your own words, please describe any health problem(s) and related treatments your dog has been to a veterinary clinic(s) for since the last survey (in November/December 2020) (veterinary treated)	N/a	Free text response
Owner expectations of veterinary costs	Compared to my expectations when I first acquired my dog, the amount I have spent at veterinary clinic(s) on my dog's health since the last survey (in November/December 2020) has been...<br>N.B. If you would like to explain your answer further in your own words, please feel free to do so below.	N/a	Less than I expected<br>As I expected<br>More than I expected<br>I'm not sure/I can't remember

File S2

Table S2. **Variables used for univariable analysis of the outcomes of total number of owner-reported disorder groups per dog (all disorders). † Categorical variable, binary. ‡Categorical variable, more than two categories. *Continuous variable.**

Health Outcome		Variable	Survey time point
Total number of owner-reported disorder groups per dog (all disorders) (continuous variable)	Information theory	† Owner gender	2020
		‡Owner age	2020
		† First-time owner	2020
		† Dog neuter status	2020, updated 2021
		† Dog sex	2020
		† Insurance status	2020, updated 2021
		Breed (top 12 breeds)	2020
		‡Breed purity categorisation	2020
		‡UK Kennel Club breed group	2020
		‡Breed designation (pure, cross, designer)	2020
		‡Typical adult bodyweight categorisation	2020
	Illegal sale indicators	† Sold without microchip details	2020
		† Sold less than 6 weeks of age	2020
		† Sold without seeing mum	2020
		† Collection outside breeders property	2020
		† Sold < 13 weeks of age with a passport	2020
	Owner/demographics	† Works in animal sector	2020
	Purchase behaviours	† Purchased via animal selling website	2020
		† Viewed in person prior to purchase	2020
		† Picked breeder as did health tests	2020
		† Given health check by vet	2020
		† Asked to put down deposit	2020
		† Asked to see DNA tests	2020
		† Asked to see health screen results	2020
		† Breeder provided vaccination details	2020
		† Breeder gave diet advice	2020
		† Breeder gave health advice	2020

	† Breeder gave exercise advice	2020
	† Breeder wormed puppy	2020
	† Breeder provide flea treatment	2020
	† Breeder gave first vaccinations	2020
	† Breeder gave second vaccinations	2020
Health at time of survey	† Current health issues	2020
	† Disorder count binary soon after brought puppy home	2020
	† Been to vet since brought puppy home	2020
Purchase motivations for a dog/breed or crossbreed chosen	† Healthy breed	2020
	† Size suited to lifestyle	2020
	† Hypoallergenic	2020
	† Long life expectancy	2020

Table S3. **Variables used for univariable analysis of owner expectations of veterinary costs. † Categorical variable, binary. ‡Categorical variable, more than two categories. *Continuous variable.**

Health Outcome	Variable Category	Variable	Survey time point
Owner expectations of veterinary costs (binary categorical variable)	Information theory	† Owner gender	2020
		‡Owner age	2020
		† First-time owner	2020
		† Dog neuter status	2020, updated 2021
		† Dog sex	2020
		† Insurance status	2020, updated 2021
		‡Breed (top 12 breeds)	2020
		‡Breed purity categorisation	2020
		‡UK Kennel Club breed group	2020
		‡Breed designation (pure, cross, designer)	2020
		‡Typical adult bodyweight categorisation	2020
	Illegal sale indicators	† Sold without microchip details	2020
		† Sold less than 6 weeks of age	2020
		† Sold without seeing mum	2020
		† Collection outside breeders property	2020
		† Sold < 13 weeks of age with a passport	2020
	Owner/demographics	† Works in animal sector	2020
		‡Living situation	2020
		†Living with kids	2020
	Pre-purchase research	† Research before buying	2020
		*Expected puppy/dog to costs per year	2020
	Purchase behaviours	† Purchased via animal selling website	2020
		† Purchased via Kennel Club	2020
		† Viewed in person prior to purchase	2020
		† Picked breeder as did health tests	2020
		† Given health check by vet	2020
		† Asked to put down deposit	2020
		† Breeder provided vaccination details	2020

	† Reasonably priced puppies	2020
	*Price of puppy	2020
	† Asked to see DNA tests	2020
	† Asked to see health screen results	2020
	† Expectations of breeder - member Kennel Club Assured Breeder Scheme	2020
	† Dog bred from won awards	2020
	† Breeder gave diet advice	2020
	† Breeder gave health advice	2020
	† Breeder gave exercise advice	2020
	† Breeder wormed puppy	2020
	† Breeder provide flea treatment	2020
	† Breeder gave first vaccinations	2020
	† Breeder gave second vaccinations	2020
	† Breeder puppy contract	2020
Health at time of survey	† Current health issues	2020
	† Disorder count binary soon after brought puppy home	2020
	† Been to vet since brought puppy home	2020
Purchase motivations for a dog/breed or crossbreed chosen	† Healthy breed	2020
	† Friends or family	2020
	† Size suited to lifestyle	2020
	† Hypoallergenic	2020
	† Affordable purchase cost of puppies	2020
	† Affordable cost of upkeep	2020
	† Low grooming needs	2020
	† Long life expectancy	2020
	† Popularity of the breed	2020
	† Encourage exercise	2020
	† Low exercise need	2020
	† Owned breed before	2020

File S3

Table S4. Final multivariable generalised linear model for the number of visits to veterinary clinics or home visits from a veterinary professional between the first questionnaire in puppyhood (November/December 2020 aged ≤ 14 months) and turning 21-months of age (between January to August 2022) amongst a cohort of UK Pandemic Puppies acquired < 16 weeks (*n* = 882).
†Included as per information theory as a variable of *a priori* interest, regardless of significance. ‡Dog demographic variables individually used to replace breed (most common 12 breeds at 21-months) in the original model. °Retained in model for improved model fit. *95% confidence interval. Significant results are emboldened.

Variable	Category	Coefficient	Std. Error	95% CI*	*p*-Value
†First-time dog owner	No			Ref	
	**Yes**	**0.45**	**0.17**	**0.11 – 0.78**	**0.01**
Owner reported they had taken their dog to the vet for any health problems since they brought them home (at time of first questionnaire)	No				
	**Yes**	**0.50**	**0.17**	**0.17 – 0.82**	**0.003**
†‡Typical adult bodyweight	≤ 10 kg	-0.15	0.20	-0.55 – 0.25	0.456
	10 to < 20 kg			Ref	**0.013**
	20 to < 30 kg	0.12	0.19	-0.26 – 0.50	0.542
	**30 to < 40 kg**	**0.64**	**0.23**	**0.19 – 1.09**	**0.006**
	≥ 40 kg	-0.72	0.57	-1.85 – 0.41	0.21
†Dog insurance status at 21-months	Not insured			Ref	
	Insured	0.39	0.22	-0.05 – 0.83	0.081
†Dog sex	Male			Ref	
	Female	-0.09	0.15	-0.38 – 0.20	0.529
†Breed	Crossbred			Ref	0.382
	Border Collie	-0.23	0.54	-1.30 – 0.83	0.668
	Border Terrier	-0.06	0.56	-1.16 – 1.04	0.922
	Cavapoo	-0.66	0.63	-1.90 – 0.58	0.297
	Cockapoo	-0.02	0.43	-0.86 – 0.83	0.97
	Cocker Spaniel	0.07	0.45	-0.81 – 0.96	0.875
	English Springer Spaniel	0.01	0.60	-1.16 – 1.19	0.982

	Golden Retriever	0.32	0.55	-0.77 – 1.41	0.561
	Labradoodle	0.39	0.55	-0.68 – 1.46	0.472
	Labrador Retriever	0.39	0.42	-0.44 – 1.21	0.36
	Miniature Smooth-Haired Dachshund	0.28	0.55	-0.81 – 1.36	0.616
	Other	0.43	0.37	-0.30 – 1.16	0.249
	Whippet	0.98	0.60	-0.19 – 2.15	0.101
†Owner age	18 – 24 years	-0.02	0.40	-0.80 – 0.77	0.967
	25 – 34 years	-0.04	0.22	-0.47 – 0.40	0.876
	35 – 44 years	0.04	0.22	-0.38 – 0.47	0.851
	45 – 54 years			Ref	0.975
	55 – 64 years	-0.12	0.22	-0.56 – 0.31	0.58
	65 – 74 years	-0.15	0.27	-0.67 – 0.37	0.57
	≥ 75 years	0.35	0.60	-0.82 – 1.52	0.56
†Owner gender	Male			Ref	
	Female	-0.08	0.26	-0.58 – 0.42	0.746
†Dog Neutered	No			Ref	
	Yes	-0.21	0.16	-0.52 – 0.09	0.175
†‡Purebred status	Crossbred			Ref	
	Purebred	0.27	0.17	-0.06 – 0.60	0.106
†‡Purebred designer status	Crossbred			Ref	0.257
	Designer Crossbred	0.13	0.38	-0.62 – 0.88	0.738
	Purebred	0.38	0.37	-0.34 – 1.10	0.304
†‡Breed group	Not KC recognised			Ref	0.412
	Gundog	0.25	0.20	-0.14 – 0.63	0.207
	Hound	0.21	0.28	-0.34 – 0.75	0.456
	Pastoral	-0.01	0.32	-0.63 – 0.61	0.978
	Terrier	0.38	0.28	-0.18 – 0.93	0.187
	Toy	0.02	0.46	-0.87 – 0.92	0.961
	Utility	0.35	0.30	-0.24 – 0.94	0.247
	Working	0.91	0.41	0.11 – 1.70	0.025
	No			Ref	

°Found the breeder of the dog/puppy via an animal specific selling website, e.g., Pets4Homes, Champdogs	Yes	-0.01	0.15	-0.31 – 0.29	0.956
°Breeder provided puppy with first vaccinations prior to acquisition	No			Ref	
	Yes	0.10	0.16	-0.21 – 0.42	0.518
°Owner paid deposit to secure puppy	No			Ref	
	Yes	0.31	0.17	-0.02 – 0.65	0.065
°Owner reported health issues they were concerned about (at time of first questionnaire)	No			Ref	
	Yes	0.64	0.37	-0.08 – 1.36	0.081

Table S5. Final multivariable generalised linear model for owner-reported approximate total veterinary care costs since the first questionnaire in puppyhood (aged ≤ 14 months) amongst a cohort of UK Pandemic Puppies acquired < 16 weeks (*n* = 408). †Included as per information theory as a variable of *a priori* interest, regardless of significance. ‡Dog demographic variables individually used to replace breed (most common 12 breeds at 21-months) in the original model. °Retained in model for improved model fit. *95% confidence interval. Significant results are emboldened.

Variable	Category	Coefficient	Std. Error	95% CI*	*p*-Value
†Dog insurance status at 21-months	Not insured			Ref	
	**Insured**	**0.27**	**0.12**	**0.03 – 0.51**	**0.029**
†‡Typical adult bodyweight	≤ 10 kg	-0.11	0.10	-0.30 – 0.08	0.251
	10 to < 20 kg			Ref	**0.022**
	20 to < 30 kg	0.05	0.09	-0.13 – 0.23	0.602
	**30 to < 40 kg**	**0.22**	**0.10**	**0.02 – 0.41**	**0.028**
	≥ 40 kg	-0.47	0.32	-1.09 – 0.15	0.14
†Owner age	18 – 24 years	0.07	0.17	-0.26 – 0.40	0.689
	25 – 34 years	0.06	0.10	-0.14 – 0.25	0.578
	35 – 44 years	0.17	0.10	-0.02 – 0.36	0.074
	45 – 54 years			Ref	**0.042**
	55 – 64 years	0.10	0.11	-0.11 – 0.32	0.341
	65 – 74 years	-0.07	0.14	-0.34 – 0.21	0.628
	**≥ 75 years**	**-1.10**	**0.40**	**-1.89 – (-0.30)**	**0.007**
†Dog sex	Male			Ref	
	Female	-0.05	0.07	-0.18 – 0.09	0.523
†Breed	Crossbred			Ref	0.233
	Labradoodle	0.13	0.28	-0.42 – 0.67	0.645
	Miniature Smooth-Haired Dachshund	-0.38	0.28	-0.93 – 0.17	0.173
	Border Collie	0.03	0.33	-0.61 – 0.68	0.918
	Cavapoo	-0.15	0.37	-0.87 – 0.57	0.685
	Cockapoo	-0.33	0.24	-0.79 – 0.14	0.166
	Cocker Spaniel	-0.29	0.24	-0.76 – 0.18	0.232
	Other	-0.08	0.21	-0.48 – 0.33	0.712

	Whippet	0.26	0.27	-0.27 – 0.80	0.335
	Labrador Retriever	0.01	0.22	-0.43 – 0.44	0.979
	Border Terrier	-0.20	0.29	-0.77 – 0.37	0.498
	Golden Retriever	0.03	0.27	-0.50 – 0.56	0.916
	English Springer Spaniel	-0.02	0.28	-0.57 – 0.54	0.955
†First-time dog owner	No			Ref	
	Yes	-0.03	0.08	-0.18 – 0.12	0.673
†Owner gender	Male			Ref	
	Female	-0.07	0.13	-0.32 – 0.18	0.567
†Dog Neutered	No			Ref	
	Yes	-0.06	0.07	-0.21 – 0.08	0.378
†‡Purebred status	Crossbred			Ref	
	Purebred	0.06	0.08	-0.10 – 0.22	0.483
†‡Purebred designer status	Crossbred			Ref	0.650
	Designer Crossbred	-0.13	0.21	-0.55 – 0.29	0.543
	Purebred	-0.06	0.21	-0.46 – 0.35	0.779
†‡Breed group	Not KC recognised			Ref	0.927
	Gundog	0.05	0.09	-0.14 – 0.23	0.625
	Hound	0.01	0.12	-0.24 – 0.25	0.971
	Pastoral	0.01	0.17	-0.32 – 0.34	0.933
	Terrier	-0.04	0.14	-0.31 – 0.23	0.786
	Toy	-0.05	0.22	-0.49 – 0.39	0.826
	Utility	0.19	0.14	-0.09 – 0.47	0.181
	Working	0.07	0.18	-0.29 – 0.43	0.693
°Purchase price of puppy	< £500			Ref	0.096
	£500 – £999	0.72	0.28	0.18 – 1.27	0.009
	£1000 – £1499	0.67	0.27	0.13 – 1.20	0.016
	£1500 – £1999	0.75	0.27	0.21 – 1.28	0.006
	≥ £2000	0.68	0.27	0.15 – 1.22	0.013
°Sold without microchip details	No			Ref	
	Yes	0.13	0.40	-0.66 – 0.92	0.747
	No			Ref	

°Breeder provided puppy with first vaccinations prior to acquisition	Yes	0.15	0.08	-0.01 – 0.30	0.059
°Breeder provided Puppy Contract	No			Ref	
	Yes	0.07	0.07	-0.07 – 0.20	0.332
°Purchased breed/crossbreed due to its popularity	No			Ref	
	Yes	0.24	0.17	-0.10 – 0.57	0.163
°Sold outside breeder's home	No			Ref	
	Yes	-0.13	0.07	-0.28 – 0.01	0.062
°Owner reported health issues they were concerned about (at time of first questionnaire)	No			Ref	
	Yes	0.23	0.15	-0.06 – 0.52	0.118

File S4.

Table S6. Prevalence of at least one episode per category of owner-reported health disorders groups that required veterinary care between the first questionnaire in puppyhood aged ≤ 7 months (November/December 2020) and turning 21 months of age (between January to August 2022) in a cohort of UK Pandemic Puppies acquired < 16 weeks of age (*n* = 943).

Disorder group categories listed in descending order of frequency. *CI confidence interval.

Grouped-level disorder	Count	Prevalence (%)	95% CI*
Enteropathy	170	18.03	15.57 – 20.48
Traumatic injury	97	10.29	8.35 – 12.23
Ophthalmological disorder	78	8.27	6.51 – 10.03
Ear disorder	68	7.21	5.56 – 8.86
Musculoskeletal disorder	64	6.79	5.18 – 8.39
Skin disorder	64	6.79	5.18 – 8.39
Intoxication	55	5.83	4.34 – 7.33
Upper respiratory tract	37	3.92	2.69 – 5.16
Foreign body	32	3.39	2.24 – 4.55
Mass lesion	22	2.33	1.37 – 3.30
Admit non-routine care	18	1.91	1.04 – 2.78
Claw nail disorder	15	1.59	0.79 – 2.39
Appetite finding	14	1.48	0.71 – 2.26
Female reproductive abnormality	14	1.48	0.71 – 2.26
Urinary tract disorder	14	1.48	0.71 – 2.26
Behaviour disorder	13	1.38	0.63 – 2.12
Dental disorder	13	1.38	0.63 – 2.12
Parasite infestation	8	0.85	0.43 – 1.67
Anal sac disorder	7	0.74	0.36 – 1.52
Neoplasia	7	0.74	0.36 – 1.52

Abscess	6	0.64	0.29 – 1.38
Hernia	6	0.64	0.29 – 1.38
Lethargy	6	0.64	0.29 – 1.38
Brain disorder	5	0.53	0.23 – 1.24
Male reproductive system	5	0.53	0.23 – 1.24
Pancreatic disorder	5	0.53	0.23 – 1.24
Thin finding	5	0.53	0.23 – 1.24
Adverse reaction to drug	4	0.42	0.17 – 1.09
Complication associated with clinical care procedure finding	4	0.42	0.17 – 1.09
Heart disease	3	0.32	0.11 – 0.93
Hepatopathy	3	0.32	0.11 – 0.93
Lower respiratory tract	3	0.32	0.11 – 0.93
Oral cavity	2	0.21	0.06 – 0.77
Abdominal finding	1	0.11	0.02 – 0.60
Disorder not diagnosed	1	0.11	0.02 – 0.60
Haematopoietic system	1	0.11	0.02 – 0.60
Infection bacterial	1	0.11	0.02 – 0.60
Lymph node	1	0.11	0.02 – 0.60
Renal disease	1	0.11	0.02 – 0.60
Vertebral spinal	1	0.11	0.02 – 0.60
Viral infectious	1	0.11	0.02 – 0.60
Weight body condition	1	0.11	0.02 – 0.60

Table S7. Prevalence of at least one episode per category of owner-reported health disorders groups requiring ongoing monitoring/treatment in 21 month old dogs from a cohort of UK Pandemic Puppies acquired < 16 weeks of age (*n* = 936).

Disorder group categories listed in descending order of frequency. *CI confidence interval.

Disorder group	Count	Prevalence (%)	95% CI*
Skin disorder	28	2.99	1.90 – 4.08
Musculoskeletal disorder finding	13	1.39	0.64 – 2.14
Behaviour disorder	8	0.85	0.43 – 1.68
Ear disorder	7	0.75	0.36 – 1.54
Enteropathy	4	0.43	0.17 – 1.09
Ophthalmological disorder finding	3	0.32	0.11 – 0.94
Anal sac disorder	2	0.21	0.06 – 0.78
Brain disorder	2	0.21	0.06 – 0.78
Dental disorder finding	2	0.21	0.06 – 0.78
Hematopoietic system finding	2	0.21	0.06 – 0.78
Mass lesion finding	2	0.21	0.06 – 0.78
Renal disease	2	0.21	0.06 – 0.78
Urinary system disorder finding	2	0.21	0.06 – 0.78
Appetite finding	1	0.11	0.02 – 0.60
Claw nail disorder	1	0.11	0.02 – 0.60
Complication clinical care procedure	1	0.11	0.02 – 0.60
Female reproductive abnormality	1	0.11	0.02 – 0.60
Heart disease	1	0.11	0.02 – 0.60
Immune mediated disease	1	0.11	0.02 – 0.60
Intoxication	1	0.11	0.02 – 0.60
Neoplasia	1	0.11	0.02 – 0.60
Pancreatic disorder finding	1	0.11	0.02 – 0.60
Spinal cord finding	1	0.11	0.02 – 0.60

Upper respiratory tract finding	1	0.11	0.02 – 0.60

Table 8. Prevalence of at least one episode per category of owner-reported health disorders in dogs (all disorders), regardless of whether veterinary advice was sought, between the first questionnaire in puppyhood (November/December 2020 aged ≤ 7 months) and turning 21 months of age (between January to August 2022) in a cohort of UK Pandemic Puppies acquired < 16 weeks of age (n = 931).

*CI confidence interval. †Includes multiple choice question (MCQ) option(s) and re-categorised free-text grouped together, with options described. Brackets refer to options from MCQ question mapped to the grouped-level disorder.

Grouped-level disorder	Count	Prevalence (%)	95% CI*
†Enteropathy (diarrhoea; eating-non food items; vomiting)	702	75.40	72.54 – 78.06
†Skin disorder (hair loss; wounds/sores on skin; itching/licking)	249	26.75	24.00 – 29.68
†Ophthalmological disorder finding (runny eye)	234	25.13	22.45 – 28.02
†Upper respiratory tract (coughing)	172	18.47	16.11 – 21.10
†Ear disorder (sore or infected ears)	164	17.62	15.30 – 20.19
†Parasite infestation (worms; fleas)	100	10.74	8.91 – 12.89
†Musculoskeletal disorder (arthritis; lameness)	98	10.53	8.72 – 12.66
†Anal sac disorder (problems with anal glands)	91	9.77	8.03 – 11.85
†Claw nail disorder (overgrown nails)	64	6.87	5.42 – 8.68
†Mass lesion finding (lumps)	38	4.08	2.99 – 5.55
†Thin (weight loss)	34	3.65	2.63 – 5.06
†Dental disorder (dental problems)	24	2.58	1.74 – 3.81
Intoxication poisoning	16	1.72	1.06 – 2.77
Traumatic injury	15	1.61	0.98 – 2.64
†Obesity (being overweight)	14	1.50	0.90 – 2.51
†Incontinence (leaking urine)	13	1.40	0.82 – 2.37
Behaviour disorder	12	1.29	0.74 – 2.24
†Brain disorder (suspected/confirmed fits/seizures)	10	1.07	0.58 – 1.97
Drug therapy	8	0.86	0.44 – 1.69
Female reproductive abnormality	8	0.86	0.44 – 1.69
Foreign body	8	0.86	0.44 – 1.69
†Lower respiratory tract finding (breathing problems)	7	0.75	0.36 – 1.54

Appetite finding	5	0.54	0.23 – 1.25
Adverse reaction to drug	4	0.43	0.17 – 1.10
Complication clinical care	3	0.32	0.11 – 0.94
Oral cavity finding	3	0.32	0.11 – 0.94
Dietary indiscretion	2	0.21	0.06 – 0.78
Heart disease	2	0.21	0.06 – 0.78
Neoplasia	2	0.21	0.06 – 0.78
Urinary system disorder	2	0.21	0.06 – 0.78
Abscess	1	0.11	0.02 – 0.61
Hernia	1	0.11	0.02 – 0.61
Renal disease	1	0.11	0.02 – 0.61
Tail disorder	1	0.11	0.02 – 0.61

File S5

Table 9: **Univariable generalised linear mixed model analysis of variables for association with the number of 'all disorders' reported in 21-month old dogs between the first questionnaire in puppyhood (aged ≤ 7 months, in November/December 2020) and dogs aged 21-months (January to August 2022) amongst a cohort of UK Pandemic Puppies acquired < 16 weeks (*n* = 841 due to missing data across different categories).**
‡Dog demographic variables individually used to replace breed (most common 12 breeds at 21-months) in the original model. *95% confidence interval for the coefficient. Results liberally associated with the outcome variable ($p \leq 0.2$) shown in bold.

	Variable	Category	Coefficient	Std. Error	95% CI*	*p*-Value
**Information theory**	Owner gender	Female			Ref	
		**Male**	**-0.38**	**0.17**	**-0.71 – (-0.05)**	**0.02**
	Owner age	18 – 24 years	0.44	0.26	-0.06 – 0.95	0.09
		25 – 34 years	0.14	0.14	-0.14 –0.42	0.32
		35 – 44 years	-0.04	0.14	-0.32 –0.25	0.81
		**45 – 54 years**			**Ref**	**<0.001**
		55 – 64 years	-0.38	0.15	-0.66 – (-0.09)	0.01
		65 – 74 years	-0.60	0.18	-0.95 – (-0.26)	<0.001
		≥ 75 years	-0.44	0.40	-1.23 –0.35	0.28
	First-time dog owner	No			Ref	
		**Yes**	**0.59**	**0.10**	**0.40 – 0.78**	**<0.001**
	Dog Neutered	No			Ref	
		**Yes**	**0.13**	**0.10**	**-0.07 –0.32**	**0.20**
	Dog sex	Female			**Ref**	
		**Male**	**-0.24**	**0.10**	**-0.43 – (-0.05)**	**0.01**
	Dog insurance status at 21-months	Not insured			Ref	
		**Insured**	**0.36**	**0.15**	**0.07 –0.65**	**0.01**
	Breed	Crossbred			Ref	0.348
		Border Collie	-0.21	0.35	-0.90 –0.49	0.56
		Border Terrier	-0.24	0.38	-0.98 –0.50	0.52

	Cavapoo	0.06	0.42	-0.77 –0.89	0.89
	Cockapoo	0.22	0.29	-0.34 – 0.78	0.45
	Cocker Spaniel	0.30	0.30	-0.30 –0.89	0.33
	English Springer Spaniel	0.30	0.41	-0.50 –1.10	0.47
	Golden Retriever	0.09	0.37	-0.63 – 0.81	0.80
	Labradoodle	0.27	0.37	-0.45 –0.99	0.46
	Labrador Retriever	0.30	0.28	-0.26 – 0.85	0.29
	Miniature Smooth-Haired Dachshund	0.05	0.36	-0.66 –0.77	0.89
	Other	0.24	0.25	-0.25 –0.72	0.34
	Whippet	0.99	0.40	0.22 – 1.77	0.01
‡Purebred status	Crossbred			Ref	
	Purebred	0.09	0.11	-0.12 – 0.30	0.40
‡Breed group	Not KC recognised			Ref	0.36
	Gundog	0.18	0.12	-0.07 –0.42	0.15
	Hound	0.14	0.18	-0.21 –0.50	0.43
	Pastoral	-0.18	0.21	-0.58 –0.23	0.39
	Terrier	-0.13	0.18	-0.50 –0.23	0.47
	Toy	0.37	0.31	-0.24 –0.97	0.24
	Utility	0.09	0.20	-0.31 –0.48	0.66
	Working	0.34	0.27	-0.19 –0.86	0.21
‡Purebred/designer status	Crossbred			Ref	0.55
	Designer Crossbred	0.18	0.26	-0.33 –0.68	0.50
	Purebred	0.24	0.25	-0.24 –0.72	0.33
‡Typical adult bodyweight	≤ 10 kg	-0.27	0.13	-0.53 –0.00	0.05
	**10 to < 20 kg**			**Ref**	**0.04**
	20 to < 30 kg	0.02	0.13	-0.23 –0.28	0.86
	30 to < 40 kg	0.25	0.15	-0.04 –0.55	0.86
	≥ 40 kg	0.23	0.38	-0.52 –0.97	0.55
**Illegal sale indicators**	No			Ref	

	Sold without microchip details	**Yes**	**0.61**	**0.40**	**-0.18 – 1.39**	**0.13**
	Sold under six weeks of age	No			Ref	
		**Yes**	**3.74**	**1.05**	**1.69 – 5.80**	**<0.001**
	Sold without mother present	No			Ref	
		**Yes**	**0.39**	**0.12**	**0.15 – 0.62**	**0.001**
	Collected puppy outside breeder property	No			Ref	
		Yes	0.10	0.10	-0.10 – 0.30	0.33
	Sold < 13 weeks of age with a passport	No			Ref	
		Yes	-0.19	0.22	-0.63 – 0.24	0.38
**Owner/demographics**	Owner employed in the canine and/or animal care sector (e.g. veterinary nurse, dog groomer, dog trainer, etc.)	No			Ref	
		Yes	0.15	0.19	-0.23 – 0.52	0.45
Purchase behaviours	Owner found breeder via an animal specific selling website, e.g., Pets4Homes, Champdogs	No			Ref	
		Yes	0.03	0.10	-0.16 – 0.22	0.75
	Owner visited the breeder's property in person prior to bringing puppy home	No			Ref	
		Yes	0.10	0.11	-0.10 – 0.31	0.33
	Owner saw health test results of mother/father	No			Ref	
		**Yes**	**0.19**	**0.10**	**-0.01 – 0.38**	**0.06**
	Breeder provided health check by a vet	No			Ref	
		Yes	0.16	0.16	-0.17 – 0.48	0.34
	Owner paid deposit to secure puppy	No			Ref	
		**Yes**	**0.22**	**0.11**	**0.01 – 0.44**	**0.04**
		No			Ref	

	Owner asked breeder for results of DNA (genetic) tests	Yes	0.04	0.10	-0.16 – 0.24	0.69
	Owner picked breeder as they conducted health tests	No			Ref	
		Yes	0.02	0.10	-0.18 – 0.22	0.84
	Breeder provided puppy's vaccinations record	No			Ref	
		Yes	0.14	0.23	-0.31 – 0.59	0.55
	Breeder provided advice on puppy's diet	Yes			Ref	
		No	-0.09	0.12	-0.32 – 0.15	0.47
	Breeder provided advice on puppy's health	No			Ref	
		Yes	0.03	0.10	-0.16 – 0.23	0.76
	Breeder provided advice on puppy's exercise regime	No			Ref	
		Yes	-0.10	0.10	-0.30 – 0.10	0.34
	Breeder gave worming treatment before owner took puppy home	No			Ref	
		**Yes**	**-0.34**	**0.17**	**-0.68 – 0.00**	**0.05**
	Breeder provided flea treatment	No			Ref	
		Yes	-0.13	0.10	-0.33 – 0.08	0.23
	Breeder provided first vaccinations	No			Ref	
		Yes	0.10	0.10	-0.11 – 0.31	0.34
	Breeder provided second vaccinations	No			Ref	
		Yes	-0.13	0.23	-0.57 – 0.32	0.57
Health at time of survey	Owner reported health issues they were concerned about (at time of first questionnaire)	No			Ref	
		**Yes**	**1.02**	**0.24**	**0.55 – 1.49**	**<0.001**
	Owner reported dog had one or more disorders 'soon after' acquisition	No			Ref	
		**Yes**	**0.60**	**0.12**	**0.37 – 0.83**	**<0.001**
		No			Ref	

	Owner took dog to a vet for any health problem(s) after they brought them home (at the time of the first questionnaire)	**Yes**	**0.35**	**0.11**	**0.14 – 0.56**	**0.001**
Purchase motivations for a dog/breed or crossbreed chosen	Looking for characteristic of generally healthy breed/crossbreed when selecting a particular breed/crossbreed to buy	No			Ref	
		Yes	0.00	0.10	-0.19 – 0.19	0.99
	Looking for characteristic of size suited to lifestyle when selecting a particular breed/crossbreed to buy	No			Ref	
		Yes	-0.10	0.11	-0.31 – 0.10	0.33
	Looking for characteristic of hypoallergenic when selecting a particular breed/crossbreed to buy	No			Ref	
		Yes	-0.01	0.13	-0.26 – 0.24	0.93
	Purchased breed/crossbreed due to long life-expectancy	No			Ref	
		**Yes**	**-0.26**	**0.13**	**-0.52 –0.01**	**0.05**

File S6

Table 10. Ease of access to veterinary care reported by owners of dogs aged 21 months within the UK Pandemic Puppies cohort. Owners answered for a series of scenarios to a core question of "How easy have you/your household found accessing veterinary care for your dog since the last survey in November/December 2020?".
The answer options listed represent all the choices available to respondents.

Scenario	Available respondent choices	*N*	%
Getting an appointment with my first-choice veterinary clinic (*n*=941)	Always easy	720	76.51
	Sometimes easy	142	15.09
	Rarely easy	21	2.23
	Never easy	11	1.17
	I haven't tried to make this kind of appointment	17	1.81
	I'm not sure/I can't remember	0	0.00
	Not applicable	30	3.19
Getting an appointment with any veterinary clinic for an out of hours service (e.g., evenings, weekends) (*n*=922)	Always easy	252	27.33
	Sometimes easy	57	6.18
	Rarely easy	18	1.95
	Never easy	7	0.76
	I haven't tried to make this kind of appointment	170	18.44
	I'm not sure/I can't remember	2	0.22
	Not applicable	416	45.12
Getting an appointment as quickly as I felt it was needed (*n*=926)	Always easy	625	67.49
	Sometimes easy	186	20.09
	Rarely easy	21	2.27
	Never easy	15	1.62
	I haven't tried to make this kind of appointment	34	3.67
	I'm not sure/I can't remember	0	0.00
	Not applicable	45	4.86
Getting an appointment with my preferred veterinary surgeon from my vet practice (if you have one) (*n*=923)	Always easy	255	27.63
	Sometimes easy	141	15.28
	Rarely easy	38	4.12
	Never easy	22	2.38

	I haven't tried to make this kind of appointment	148	16.03
	I'm not sure/I can't remember	3	0.33
	Not applicable	316	34.24
Getting an appointment to see any veterinary surgeon (*n*=929)	Always easy	608	65.45
	Sometimes easy	112	12.06
	Rarely easy	10	1.08
	Never easy	11	1.18
	I haven't tried to make this kind of appointment	52	5.60
	I'm not sure/I can't remember	3	0.32
	Not applicable	133	14.32
Getting an appointment to see a veterinary nurse (*n*=925)	Always easy	477	51.57
	Sometimes easy	77	8.32
	Rarely easy	7	0.76
	Never easy	4	0.43
	I haven't tried to make this kind of appointment	115	12.43
	I'm not sure/I can't remember	2	0.22
	Not applicable	243	26.27

Table 11. Veterinary access as reported by owners of UK Pandemic Puppies acquired <16 weeks, at the time of the 21-month survey in response to the question "How have you/other members of your household accessed veterinary care for your dog since the last survey in November/December 2020?".

The answer options listed represent all the choices available to respondents.

Scenario	Available respondent choices	*N*	%
I/another member of my household have been able to accompany my dog during their consultation (*n*=921)	Always	401	43.54
	Sometimes	296	32.14
	Rarely	63	6.84
	Never	157	17.05
	I'm not sure/I can't remember	4	0.43
I/another member of my household have had consultations with a veterinary surgeon/veterinary nurse via video call (e.g., Zoom, Teams, FaceTime, etc.) (*n*=895)	Always	35	3.91
	Sometimes	67	7.49
	Rarely	24	2.68
	Never	709	79.22
	I'm not sure/I can't remember	60	6.70
I/another member of my household have had consultations with a veterinary surgeon/veterinary nurse via telephone (*n*=889)	Always	60	6.75
	Sometimes	243	27.33
	Rarely	60	6.75
	Never	481	54.11
	I'm not sure/I can't remember	45	5.06
